# Rational design of caspase-responsive smart molecular probe for positron emission tomography imaging of drug-induced apoptosis

**DOI:** 10.7150/thno.35084

**Published:** 2019-09-21

**Authors:** Ling Qiu, Wei Wang, Ke Li, Ying Peng, Gaochao Lv, Qingzhu Liu, Feng Gao, Yann Seimbille, Minhao Xie, Jianguo Lin

**Affiliations:** 1 Key Laboratory of Nuclear Medicine of Ministry of Health, Jiangsu Key Laboratory of Molecular Nuclear Medicine, Jiangsu Institute of Nuclear Medicine, Wuxi, Jiangsu, China; 2 Department of Medical Imaging, Jinling Hospital, Medical School of Nanjing University, Nanjing, Jiangsu, China; 3 Department of Radiology and Nuclear Medicine, University Medical Center Rotterdam, Erasmus MC, Wytemaweg 80, 3015 CN Rotterdam, The Netherlands

**Keywords:** positron emission tomography (PET), smart molecular probe, caspase-responsive, apoptosis, rapid [18F]fluorination

## Abstract

**Purpose:** Positron emission tomography (PET) imaging of apoptosis is very important for early evaluation of tumor therapeutic efficacy. A stimuli-responsive probe based on the peptide sequence Asp-Glu-Val-Asp (DEVD), [^18^F]DEVD-Cys(StBu)-PPG(CBT)-AmBF_3_ ([^18^F]**1**), for PET imaging of tumor apoptosis was designed and prepared. This study aimed to develop a novel smart probe using a convenient radiosynthesis method and to fully examine the sensitivity and specificity of the probe response to the tumor treatment.

**Methods:** The radiolabelling precursor DEVD-Cys(StBu)-PPG(CBT)-AmBF_3_ (**1**) was synthesized through multistep reactions. The reduction together with caspase-controlled macrocyclization and self-assembly of **1** was characterized and validated *in vitro*. After [^18^F]fluorination in the buffer (pH= 2.5), the radiolabelling yield (RLY), radiochemical purity (RCP) and stability of the probe [^18^F]**1** in PBS and mouse serum were investigated by radio-HPLC. The sensitivity and specificity of [^18^F]**1** for detecting the drug-induced apoptosis was fully evaluated *in vitro* and *in vivo*. The effect of cold precursor **1** on the cell uptake and tumor imaging of [^18^F]**1** was also assessed. The level of activated caspase-3 in Hela cells and tumors with or without apoptosis induction was analyzed and compared by western blotting and histological staining.

**Results:** The whole radiosynthesis process of [^18^F]**1** was around 25 min with RLY of 50%, RCP of over 99% and specific activity of 1.45 ± 0.4 Ci/µmol. The probe was very stable in both PBS and mouse serum within 4 h. It can be activated by caspase-3 and then undergo an intermolecular cyclization to form nanosized particles. The retained [^18^F]**1** in DOX-treated HeLa cells was 2.2 folds of that in untreated cells. Within 1 h microPET imaging of the untreated Hela-bearing mice, the injection of [^18^F]**1** resulted in the increase of the uptake ratio of tumor to muscle (T/M) only from 1.74 to 2.18, while in the DOX-treated Hela-bearing mice T/M increased from 1.88 to 10.52 and the co-injection of [^18^F]**1** and **1** even led to the increase of T/M from 3.08 to 14.81.

**Conclusions:** A caspase-responsive smart PET probe [^18^F]**1** was designed and prepared in a kit-like manner. Co-injection of [^18^F]**1** and 1 generated remarkably enhanced tumor uptake and signal-to-noise ratio in the tumor-bearing mice with drug-induced apoptosis, which correlated well with the expression level of activated caspase-3. This early readout of treatment response ensured that the probe [^18^F]**1** could serve as a promising PET imaging probe for timely and noninvasive evaluation of tumor therapy.

## Introduction

As for the cancer patients, early evaluation of tumor response to therapy is very important for them to decide whether the therapeutic regimen needs to be adjusted and to select the most effective treatment timely. Currently, physical measurement of tumor volume reduction is the most commonly-used approach to assess the tumor progression and therapeutic efficacy in clinics with the help of imaging techniques such as magnetic resonance imaging (MRI) and X-ray computed tomography (CT) 1, 2. However, this approach is relatively insensitive and often requires a long treatment course to visualize the tumor anatomical changes, which might result in a prolonged period of inappropriate therapy, limiting the treatment efficacy and impairing the survival rate and life quality of patients. Fortunately, these barriers to measure the early therapy response could be overcome by observing the molecular-level alterations of biomarkers that occur in dying cells after anticancer therapy 3-10. Cell death via apoptotic and necrotic pathways is known to play an important role in tissue homeostasis that is associated with several diseases and response to therapy. The degree of both apoptosis and necrosis can elucidate the mechanism of cell death and provide valuable information for evaluating the therapeutic efficacy.

The alterations of direct or indirect biomarkers for tumor cell death resulting from treatment have been identified according to a series of biochemical and molecular events 3-10. Direct imaging biomarkers include the active caspase-3 and plasma membrane depolarization. The activation of caspase-3 commits the cell to programmed cell death (apoptosis) 3, 4, while the plasma membrane depolarization 6, 7 occurs downstream of caspase-3 or is exposed as a result of membrane rupture during necrosis. Several diverse imaging techniques have been used to detect the biomarkers so as to measure and monitor the response of different types of malignant tumors to chemotherapy, radiotherapy, or hormonal therapy 3-10. Positron emission tomography (PET) has been acknowledged as an advanced noninvasive imaging technique with high sensitivity and deep tissue penetration for molecular target validation and clinical disease diagnosis in living subjects. Therefore, development of a noninvasive imaging strategy that can detect the abnormal level of apoptosis or monitor the apoptosis level induced by treatments in a variety of disease states would be of tremendous significance. Accordingly, an ideal PET imaging probe targeting apoptosis is essential for achieving such a purpose as it can noninvasively track multiple lesions simultaneously and can be used to monitor the treatment or disease progression.

In the past decade, several PET imaging probes have been developed for detecting the activity of caspase-3. For example, the utility of [^18^F]-labeled isatins to form an intracellular enzyme inhibitor complex with caspase-3 through covalent binding to the enzyme active site has been described 11, 12. Further refinement for improving the specific activity and shortening the synthesis time has also been carried out 13, 14. However, poor metabolic stability and lack of target specificity still remained the important issues of these isatin-based radiotracers 15. For example, isatin-based tracers were reported to have high background in the liver and intestines as well as low absolute tumor uptake, which might limit its routine application in clinics 14,15. Recently, caspase-3 substrate-based probes for PET imaging of apoptosis in tumors were extensively explored 16-20. [^18^F]CP18 was one of the first examples of ^18^F-labeled caspase-3 substrate radiotracers, in which a DEVD sequence served as the key recognition element and a short polyethyleneglycol (PEG) chain was introduced to obtain better pharmacokinetic properties 16-18. Accordingly, a better tumor imaging with relatively high signal-to-noise ratio was achieved. Nevertheless, it should be noted that rapid washout of small molecular probes from the target is still a big challenge for achieving a successful tumor imaging. To generate a longer retention of the probe signal in tumor cells, a novel probe [^18^F]C-SNAT based on the mechanism of caspase-3-triggered cyclization and nanoaggregation was developed 21. Taking advantage of the cleavage activity of the effector enzyme caspase-3, the peptide substrate was cleaved and an intramolecular cyclization reaction was initiated. The cyclized products are more rigid and hydrophobic, and they are susceptible to self-assembly to form nanoparticles via π-π stacking interactions *in situ*. Since the nanoparticles are difficult to be pumped out by the cells due to their bigger size and higher hydrophobicity, the retention of radioactive signal in the target would be enhanced and prolonged. This strategy significantly increased the tumor uptake and remarkably increased the tumor-to-muscle (T/M) uptake ratio in the preclinical tumor-bearing models 21-23. Similar to caspase-3, caspase-7 can also preferentially cleave the substrate following the recognition of the peptide sequence DEVD 21.

In spite of great development of radiopharmaceuticals for imaging apoptosis, no ideal probe has been approved for routine detection of cell death in the clinical application yet. As a desirable probe for monitoring apoptosis in a wide range of diseases, it should have the following unique features: 1) convenient radiosynthesis in a single-step kit manner, 2) high stability and biocompatibility, 3) high sensitivity and specificity for tumor apoptosis, and 4) high quality images within a proper time. Therefore, it remains highly challenging for scientists to design and develop desirable radiopharmaceuticals based on novel signal amplification strategies for clinical applications.

Among several *β*^+^-emitting nuclides for PET imaging, fluorine-18 is the most preferred radioisotope because of its excellent nuclear properties, such as moderate half-life (t_1/2_ = 109.8 min) and on-demand production of large quantities from a medical cyclotron 24. The decay character of fluorine-18 requires a rapid, simple and economical [^18^F]fluorination method, which has been a long-term challenge for radiochemists. Therefore, one-step aqueous radiolabelling approach based on the moiety of ammoniomethyl-trifluoroborate (AMBF_3_) would simplify the tracer development greatly since it can provide a tracer with high purity and high specific activity at a mild temperature 25-28. Such a methodology also introduced the ^18^F-labelled bioconjugates in a 'kit' form without HPLC purification, which would be quite convenient and economical for the production and application of the radiotracer in the clinical trials.

**Scheme 1:**
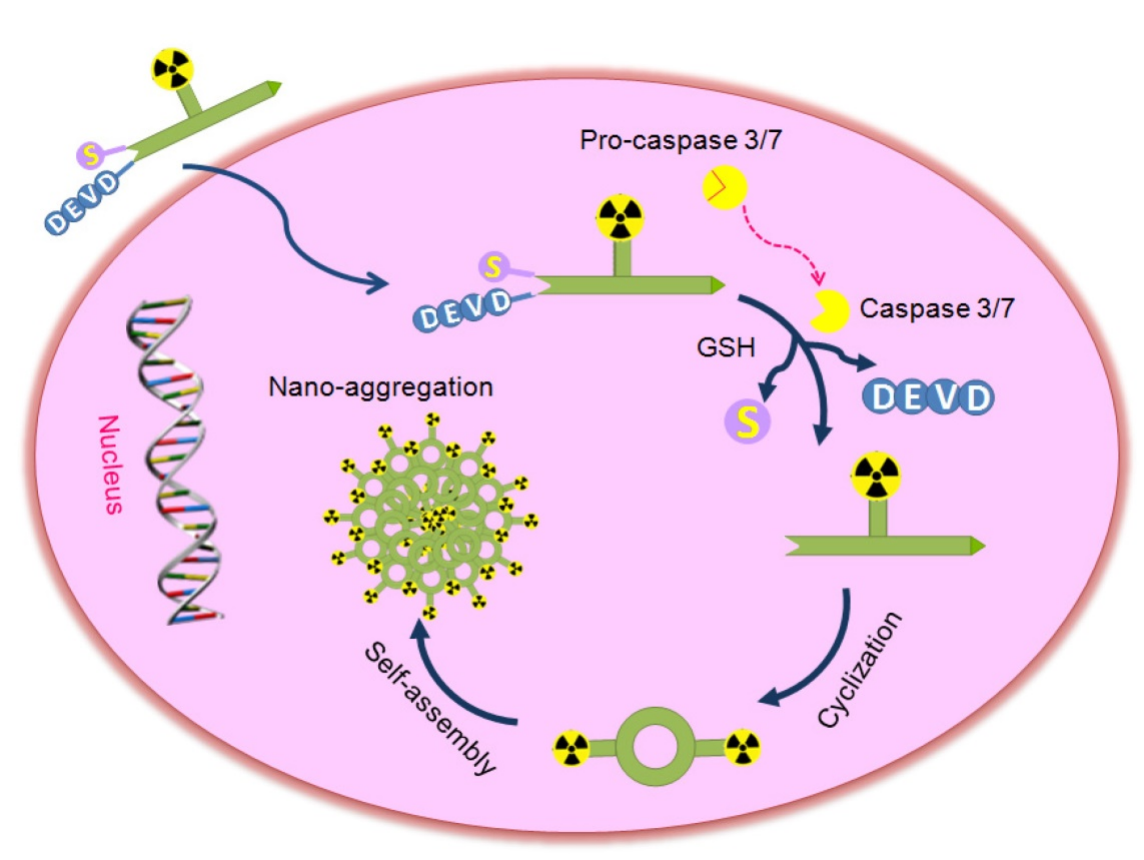
Proposed mechanism for the activatable probe [^18^F]**1** under the environment of apoptotic tumor cells. In therapy-responsive cells, increased cell membrane permeability and extensive activation of caspase-3/7 in progression to cell death permit the uptake of the probe. Cleavage of the peptide sequence DEVD and the disulfide bond yields the cyclic amphiphilic dimers, which could self-assemble into nanoparticles to generate high density of fluorine-18 activity in situ.

Inspired by previous studies, a novel smart apoptosis-specific PET imaging probe [^18^F]DEVD-Cys(StBu)-PPG(CBT)-AmBF_3_ ([^18^F]**1**) was designed in the present work based on the caspase-triggered self-assembly mechanism with a kit-like [^18^F]fluorination method (Scheme 1). This probe consists of a peptide sequence DEVD for caspase-3/7 cleavage, a disulfide-protected cysteine motif for providing the condensation group 1,2-aminothiol, and the tracer signal [^18^F]AMBF_3_ that was introduced to a propargyl-glycine (PPG) residue between cysteine and 2-cyanobenzothiazole (CBT) for PET imaging. The thiol group of cysteine in [^18^F]**1** was protected by forming a disulfide bond, not only for stabilizing the compound under physiological conditions but also for latter reduction by the reductive biothiols in tumor cells. In brief, the probe [^18^F]**1** has the ability to undergo self-assembly into nanoparticles in the tumor when it is triggered by a target enzyme of interest, which is similar to other published probes 21-23, 29-31. However, compared with the probes reported previously, the activatable probe [^18^F]**1** can be stable enough under physiological conditions due to its simple skeleton structure and it can be produced in high dose in a single run which is beneficial for the clinical application. This probe combines the advantages of small molecules for efficient penetration of cell membrane with those of nanoparticles for signal amplification, which could provide a high resolution as well as deep tissue penetration imaging of apoptosis-related physiologic processes or diseases.

## Materials and methods

### Syntheses and *in vitro* experiments

Chemical synthesis and characterization of cold probe **1** and fluorescence probe** 1-FITC** (Figures S1-S14), stability assay of **1** in pyridazine-HCl buffer (pH = 2.0-2.5) (Figure S15), *in vitro* characterization of reduction and macrocyclization of **1** (Figures S16 and S17), enzyme kinetics study of **1** (Figure S18), biocompatibility assay of **1** in cells (Figure S19), radiolabelling and quality control of probe **[^18^F]1** (Figure S20), determination of partition coefficient (log *P*), *in vitro* stability assay of **[^18^F]1** (Figure S21), western blotting analysis of caspase-3 activity in doxorubicin (DOX)-treated HeLa cells, fluorescence imaging for localization of **1** and active caspase-3 (Figure S22), cellular uptake assay of probe **[^18^F]1** (Figure S23), muscle uptake of [^18^F]**1** in DOX-treated or untreated Hela-bearing mice (Figure S24) as well as histopathologic analysis of DOX-treated tumors (Figure S25) can be found in the Supplemental Material.

### Animal model

BALB/c nude mice (18-20 g, 4-6 weeks old) were used for animal experiments. Mice were housed with free access to food and water, and allowed ample time to acclimatize before the experiments. The tumor models were established by subcutaneous injection of human cervical cancer cells HeLa (1~2×10^6^) suspended in PBS (100 µL) in the right shoulder or the posterior limb of the nude mouse. The tumors were allowed to grow for around 3-4 weeks to reach the size of 0.5-1.0 cm in diameter. Then, a subset of mice were treated with intratumoral or intravenous injection of DOX (0.2 mg, in 20 µL). Three days later, the animals were used for PET imaging and then the tumors were removed for the analysis of caspase-3 activity. All procedures were approved by the Animal Care and Ethics Committee of Jiangsu Institute of Nuclear Medicine.

### PET imaging

PET scanning was carried out using an Inveon Dedicated microPET scanner (Siemens). In all experiments, the mice were anesthetized with isoflurane (2% isoflurane in oxygen at a flow rate of 2 L/min). One group of HeLa-bearing mice were scanned at baseline (without DOX treatment) as a control. For the experimental groups, microPET imaging was performed for one group of mice three days after a single intratumoral injection of DOX (0.2 mg) and the other group was performed after intravenous injection of DOX (0.2 mg) every 4 days for a total of three administrations. The probe [^18^F]**1** (~100 μCi, in 100 μL physiological saline) was administrated by intravenous injection via the tail vein. For the co-injection group, the mice were administrated by intravenous injection of the probe [^18^F]**1** with the precursor **1** (20 µmol/kg) via the tail vein. PET images were acquired in a list mode date for 60 min. The PET data were binned into 12 frames and each frame was reconstructed with the OSEM3D/MAP algorithm using microPET Manager (version 6869, Siemens). The reconstructed pixel size was 0.78 × 0.78 × 0.80 mm on a 128 × 128 × 159 image matrix. All PET images were corrected for decay but not for attenuation. Each image was analyzed using ASIPro software (Siemens). Accurate PET quantification depends highly on precise Hounsfield units calibration, and this quantification procedure calibrates PET data to microcuries per cubic centimeter. The process is briefly as follows: 1) In IAW (Imagery Analysis Workpoint), perform a PET acquisition and an attenuation-corrected reconstruction that reflects planned scanning work; 2) In ASIPro (Acquisition Sinogram and Image Processing), designate a scale, and calculate a calibration factor for that scale within a region of interest; 3) Save the calibration factor and designated scale to the normalization file that was used to reconstruct the PET image. To characterize the accumulation of the radiotracer in the tumor, region-of-interest (ROI) analysis was performed manually by visualizing the tumor site as it appeared as bumps under the skin to identify the activity originating in the tumor region. The effects of DOX treatment and injection method were evaluated from the difference in the uptake of probe [^18^F]**1** in the control group and the experimental group.

### Histological analysis

Tumor or muscle tissues were collected on ice and washed with PBS to remove excessive blood. The tissues were fixed in 4% fresh paraformaldehyde at 4 °C for 2 h and treated sequentially with 15% and 30% sucrose until tissues were fully penetrated. Then the tissues were embedded in OCT (Sakura) and sectioned at 6 μm on cryotome (Thermo Scientific, HM 525). H&E staining was carried out according to the H&E staining kit instructions. For immunofluorescent staining, tissue sections were blocked with TBS supplemented with 0.1% triton X-100 and 5% BSA for 1 h at room temperature, followed by incubation with the cleaved caspase-3 primary antibody at 4 °C overnight. Tissue sections were washed, incubated with Cy3-labeled Goat Anti-Rabbit IgG secondary antibody for 1 h at room temperature and then counterstained with 4',6-diamidino-2-phenylindole (DAPI) for 2 min. After sealing the sections under a coverslip with anti-fade reagent, the images were acquired by Olympus IX51 fluorescence microscope.

### Western blotting analysis

At the end of microPET imaging, DOX-treated or untreated mice were killed. Tumor and muscle tissues (~100 mg) were homogenized by an electronic homogenate machine in RIPA lysis buffer (1 mL) containing protease and phosphatase inhibitors (Halt Protease Inhibitor Cocktail, Thermo Fisher Scientific Inc.). Soluble lysates were harvested by centrifugation at 12,000 rpm for 10 min at 4 °C. Protein content was measured using BCA protein assay kit and 40 μg of protein was separated with a 12% SDS-PAGE gel. After blotting to PVDF membrane, the membranes were blocked with 5% skimmed milk in tris-buffered saline (TBS, pH 7.4) for 60 min at room temperature and incubated overnight at 4 °C in buffer (TBS with 0.1% Tween-20 and 5% skimmed milk) containing different antibodies. Detection of primary antibody was performed with HRP-conjugated secondary antibody. Immunoreactive bands were visualized with Western blotting luminol reagent. Densitometric analysis was performed using Image J software.

## Results

### Design and synthesis of probe 1

An activatable PET probe was designed for detecting the activity of caspase-3/7 in tumors with amplified signal, which underwent reduction and caspase-triggered intermolecular condensation and self-assembly through the π-π stacking interactions in living subjects (Scheme 1) 21, 30-33. The apoptosis-specific imaging probe [^18^F]**1** consists of a short caspase substrate Acetyl-Asp-Glu-Val-Asp (DEVD), two pro-active moieties (a side-protected cysteine and CBT) and the raidofluorination group [^18^F]AmBF_3_ on a glycine side chain. When the small molecular probe entered into therapy-responsive tumors, the DEVD sequence and the disulfide bond would be activated and involved in the caspase-3/7-triggered cleavage and the intracellular thiol-mediated reduction, respectively. Then, larger and more hydrophobic molecules (dimers) will be formed and further self-assembled into nanoparticles in situ to result in a high density of fluorine-18 activity 34, which allows for sensitive and specific detection of tumor response to therapy.

The synthesis of the cold compound **1** was simple and convenient (Figure S1). Briefly, the peptide sequence DEVD with protecting groups was synthesized using the solid phase peptide synthesis (SPPS) method, and then coupled with the compound **A** to yield the compound **B**. Subsequently, deprotection of the compound **B** yielded the compound **C** after HPLC purification. Then AMBF_3_ was introduced to a propargyl-glycine residue between CBT and cysteine through the click reaction by employing (BimH)_3_ as the ligand. Finally, the cold probe **1** in high yield was obtained after HPLC purification (Figures S1-S11). The precursor **1** was very stable under radiolabelling conditions of pyridazine-HCl buffer (pH = 2.0-2.5) even at the high temperature of 100 °C (Figure S15).

### *In vitro* characterization of reduction and macrocyclization of 1

**Figure 1:**
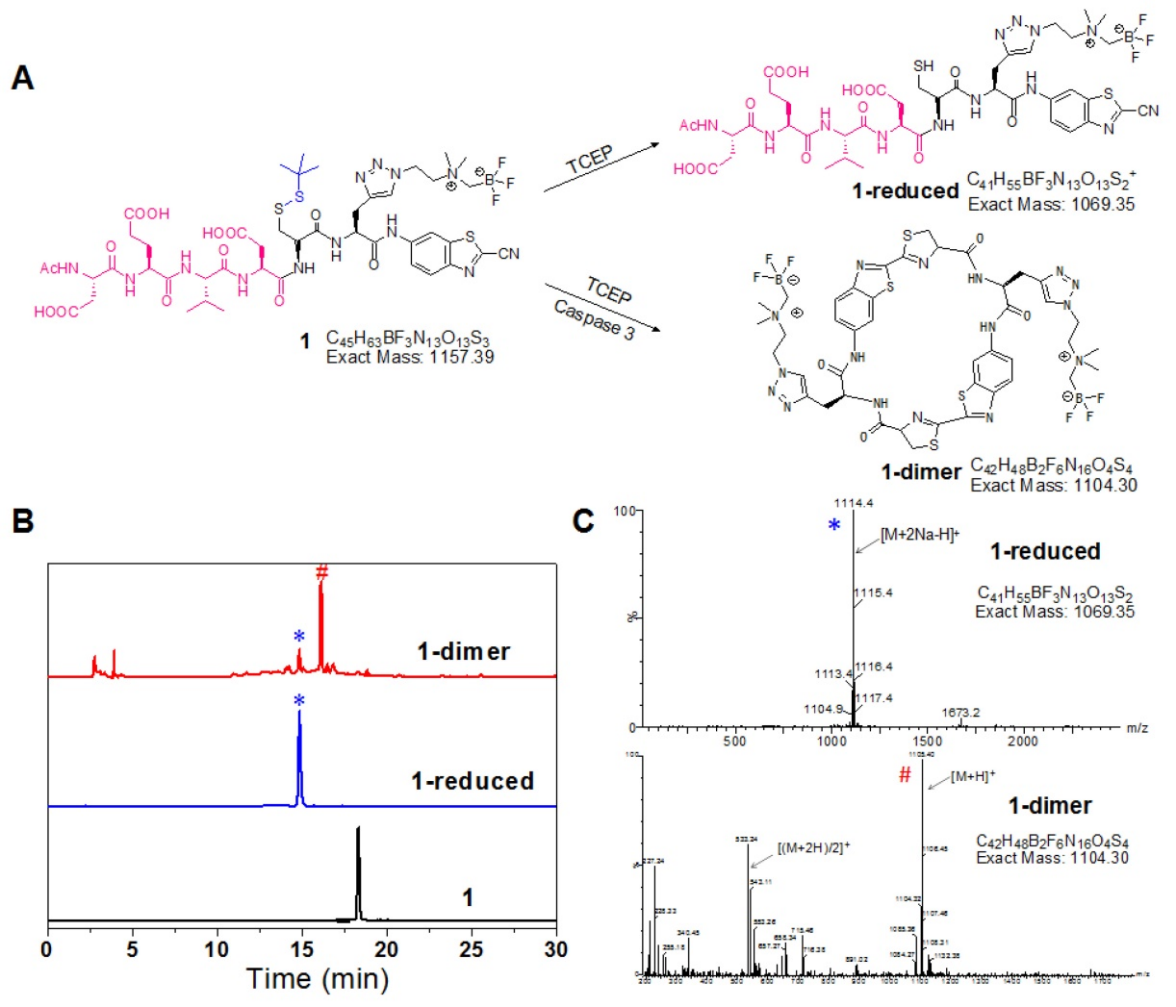
*In vitro* characterization of reduction and macrocyclization of **1**. (A) Chemical structures of the reaction products of **1** under reductive conditions or reductive and caspase-3 co-existing conditions. (B) HPLC trace of **1** incubated under reductive or reductive and caspase-3 co-existing conditions. Bottom: **1** alone; middle: reduction product **1-reduced** (*); top: cyclized product **1-dimer** (#). (C) ESI-MS characterization of the reaction products of **1** in reductive (*) and caspase-3 systems (#).

The reduction of disulfide bond in the probe **1** produced **1-reduced**, which was clearly monitored by the HPLC and LC-MS analysis (Figure 1). And the morphology of **1** after reduction and enzyme digestion has been characterized by transmission electron microscope (TEM) and dynamic light scattering (DLS) analysis (Figure S16). The established chemical reaction ensured sufficient reaction of **1** to form more rigid and hydrophobic macrocycles (**1-dimer**) with the trigger of target enzyme under physiological environment. Upon incubation with the recombinant human caspase-3 in the reaction buffer, the probe **1** was converted to **1-dimer** efficiently without obvious residual **1** observed (Figure 1). TEM and DLS analysis also demonstrated that incubation of probe 1 with the reducing agent TCEP and the enzyme caspase-3 produced monodisperse nanoparticles with the diameter at 90 ±18 nm (Figure S16A, B). In contrast, in the absence of TCEP, incubation of probe 1 with only caspase-3 in buffer produced DEVD-cleaved intermediates without detectable nanoparticles (Figure S16C, D).

To further prove the formation of **1-dimer** in apoptotic tumor cells, the content of condensed **1-dimer** was studied by incubation of probe **1** with the cell lysates of apoptotic HeLa cells. As shown in Figure S17, the peak with retention time of 16 min in HPLC trace directly indicated the formation of **1-dimer** and the content of **1-dimer** increased gradually with the extending incubation time, which was determined to be 50.99%, 57.57%, 71.68%, and 78.94% at 1, 2, 4 and 8 h, respectively (Figure S17B).

In order to evaluate the rate of probe cleavage by enzyme, the kinetic studies of probe** 1** towards caspase-3 were also performed. The Michaelis constant (*K*_m_) and turnover number (*k*_cat_) were determined to be 330.43 μM and 6.22 s^-1^, respectively (Figure S18). As a result, the *k*_cat_ /* K*_m_ value was calculated to be 17617.56 M^-1^ s^-1^. All these kinetic parameters suggested that probe **1** had sensitive response to the caspase-3.

The cytotoxicity of probe **1** and its nanoaggregates (**1-NPs**) to both cancer cell lines (HeLa) and normal cell lines (GES-1, LO2 and HEK293) was studied. As shown in Figure S19, the viability of all cell lines was determined to be higher than 90% after incubation with **1** or **1-NPs** at different concentrations (0 to 100 μM) for 3 to 24 h, respectively. This demonstrated that the cytotoxicity of both probe **1** and **1-NPs** was negligible even at the high concentration of 100 μM for 24 h.

### Radiolabelling and quality control of [^18^F]1

**Figure 2:**
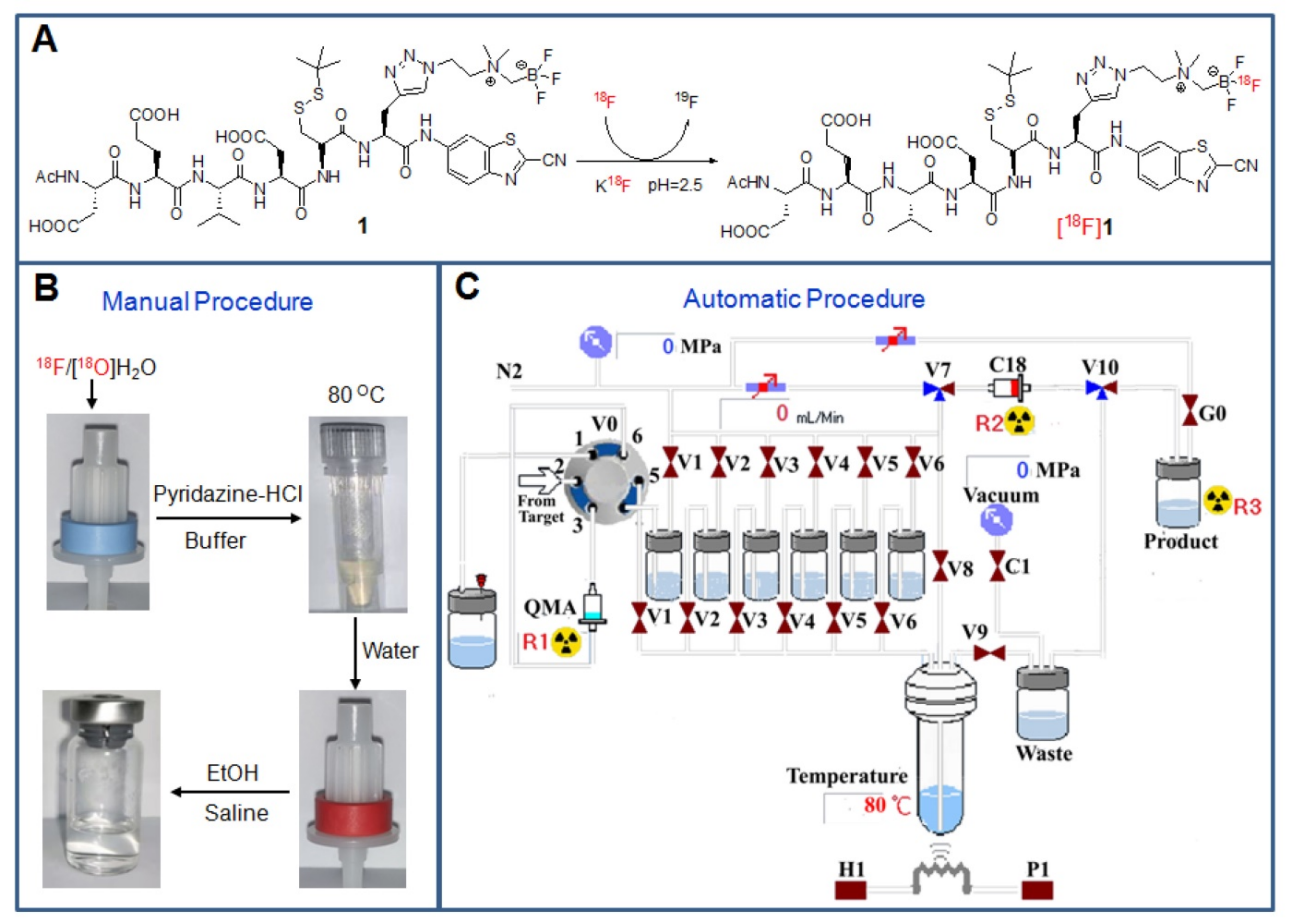
Radiosynthesis and purification of the probe [^18^F]**1**. (A) Chemical structures of the precursor** 1** and the radiotracer [^18^F]**1**. (B) Manual operation for preparing the tracer with the loading of [^18^F]fluoride onto the QMA cartridge, incubation of the reaction mixture at 80 °C, wiping off ^18^F ion on a C_18_ light cartridge and formulating the final product in saline containing <10% EtOH. (C) Automatic production of the tracer with different solutions in the vials [V1: pyridazine-HCl buffer (pH = 2.0-2.5), V2: the precursor in N,N-dimethylformamide (DMF), V3: water, V4: water, V5: ethanol, V6: saline].

Based on our previously established protocol, [^18^F]fluorination can be performed simply and easily 35. Both the manual and automatic radiosynthesis methods were suitable for the production of the tracer with similar processes (Figure 2). The total process for the radiosynthesis and purification of the tracer [^18^F]**1** only took around 25 min. The obtained radiolabelling yield (RLY) was 53 ± 6 % (decay-corrected to the end of synthesis, EOS), the radiochemical purity (RCP) was over 99% (Figure S20), and the specific activity was 1.45 ± 0.4 Ci/µmol (EOS, n=13). In addition, the radiotracer [^18^F]**1** was very stable within 4 h in both PBS and mouse serum at 37 °C (Figure S21), which would be beneficial for transportation and further *in vivo* biological studies. The octanol/water partition coefficient (log *P*) of [^18^F]**1** was determined to be -1.17 ± 0.03, indicating that this radiotracer was hydrophilic and soluble in saline for intravenous administration.

### Detection of caspase-3 activity in drug-treated cancer cells

**Figure 3:**
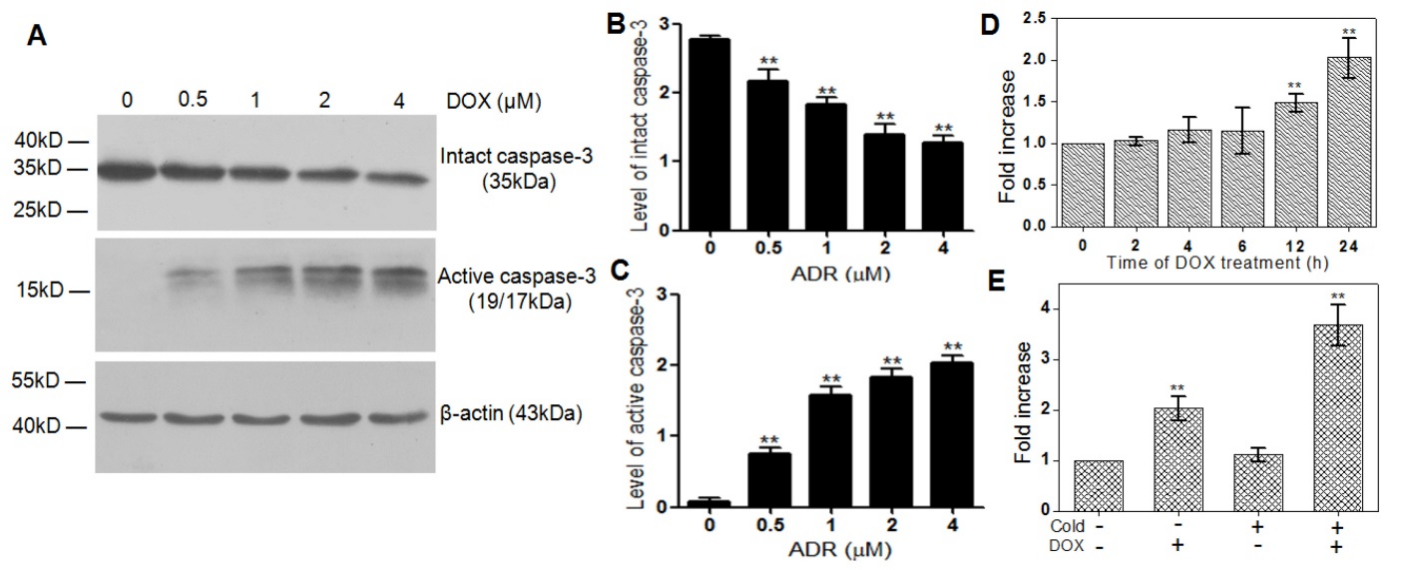
Cell uptake of [^18^F]**1** in DOX-induced apoptotic HeLa cells. (A) HeLa cells were treated with different concentrations of DOX and the changes of intact caspase-3 and active caspase-3 were detected by western blotting analysis. (B, C) Statistical analysis of western blotting results for intact caspase-3 (B) and active caspase-3 (C). (D) Uptake of [^18^F]**1** in HeLa cells treated with DOX (2 µM) for 0, 2, 4, 6, 12 and 24 h. (E) Uptake of [^18^F]**1** in HeLa cells with or without addition of cold compound **1** before and after treatment of DOX (2 µM) for 24 h. Error bars represent standard deviation (n = 3). ** p < 0.01.

The HeLa cells treated with different concentrations of DOX were chosen as the *in vitro* tumor apoptosis model and validated by western blotting analysis. As can be seen from Figure 3A, the intact caspase-3 (35 kDa) decreased significantly with the increase of the DOX concentration, while the active caspase-3 (19/17 kDa), a key apoptotic protein, increased obviously in the meantime. Compared with the DOX-untreated Hela cells, the quantified expression level of intact caspase-3 in the cells treated by 0.5, 1, 2, and 4 µM DOX decreased by 1.3, 1.5, 2.1 and 2.2 folds, respectively (Figure 3B), while that of active caspase-3 increased by 3.3, 9.3, 13.4 and 15.5 folds, respectively (Figure 3C).

To identify the subcellular localization of caspase-3 and the position of the click reaction happened in cells, the fluorescence probe **1-FITC** was synthesized with the PET signal of [^18^F]AmBF_3_ in the probe [^18^F]**1** replaced by the fluorescence signal of FITC (Figures S12-S14). As can been seen from the confocal fluorescence imaging of **1-FITC** in DOX-treated and untreated cells, DOX-treated Hela cells exhibited stronger red fluorescence than untreated Hela cells (Figure S22), which is in good accordance with the higher expression level of active caspase-3 in apoptotic tumor cells. Meanwhile, the stronger green fluorescence signal in DOX-treated tumor cells indicated the higher uptake of **1-FITC** in the treated tumor cells than that in the untreated tumor cells. Moreover, the well overlap of red and green fluorescence proved that the bioorthogonal cyclization of this probe could be triggered by the active caspase-3 and the cross-link reaction really happened at the position of caspase-3 in apoptotic cells.

Subsequently, the probe [^18^F]**1** was applied to detect the activity of caspase-3 in apoptotic HeLa cells induced by DOX (2 µM) from 0 to 24 h. As shown in Figure 3D, the retained [^18^F] activity in DOX-treated cells increased gradually with the extending time of DOX treatment, which reached 1.4 and 2.2 folds of that in naive tumor cells at 12 and 24 h, respectively. However, when the DOX-treated cells were incubated with the caspase inhibitor Z-VAD-FMK (50 µM) for 48 h before the addition of [^18^F]**1**, the retained [^18^F] activity in DOX-treated cells at 24 h decreased remarkably, which was almost identical with that in the untreated tumor cells (Figure S23). Therefore, it can be concluded that the probe [^18^F]**1** is highly sensitive and specific to the active caspase-3.

In order to substantiate the proposed trapping mechanism and further assess the effect of cold precursor **1** on the ability and sensitivity of probe [^18^F]**1** for detecting the activity of caspase-3, we studied the changes in the uptake of [^18^F]**1** in cancer cells with or without apoptosis induction and with or without addition of cold precursor** 1**. As shown in Figure 3E, the uptake of [^18^F]**1** in DOX-untreated cancer cells was almost identical in two groups with and without the addition of cold compound **1** due to the low expression level of active caspase-3 in naive tumors. However, in the DOX-treated groups, the uptake of [^18^F]**1** increased remarkably due to the high expression level of active caspase-3. Especially in the DOX-treated group with the co-incubation of [^18^F]**1** and cold precursor **1**, the cell uptake of [^18^F]**1** nearly increased by 2 folds. This was mainly due to the fact that the addition of cold precursor **1** could promote the self-condensation and assembly of [^18^F]**1** nanoparticles in situ and then lead to an enhancement in the retention of radioactivity inside tumor cells. This suggested that co-injection of [^18^F]**1** and **1** could improve the ability of [^18^F]**1** for sensitively detecting the activity of caspase-3 in apoptotic tumor cells.

### PET imaging of caspase-3 activity in drug-treated tumor

To evaluate the ability of [^18^F]**1** in monitoring the activity of caspase-3 *in vivo*, microPET imaging of nude mice bearing HeLa xenograft tumor was performed. When the tumor volume reached the size of 0.5-1.0 cm in diameter, the mice were treated with DOX (0.2 mg, 20 µL) via intratumoral injection. Three days post treatment, the radiotracer [^18^F]**1** (100-200 µCi) mixed with or without the cold precursor **1** was injected through the tail vein for PET imaging, respectively. Dynamic PET scanning was performed during the first hour post injection of the radiotracer [^18^F]**1**.

**Figure 4:**
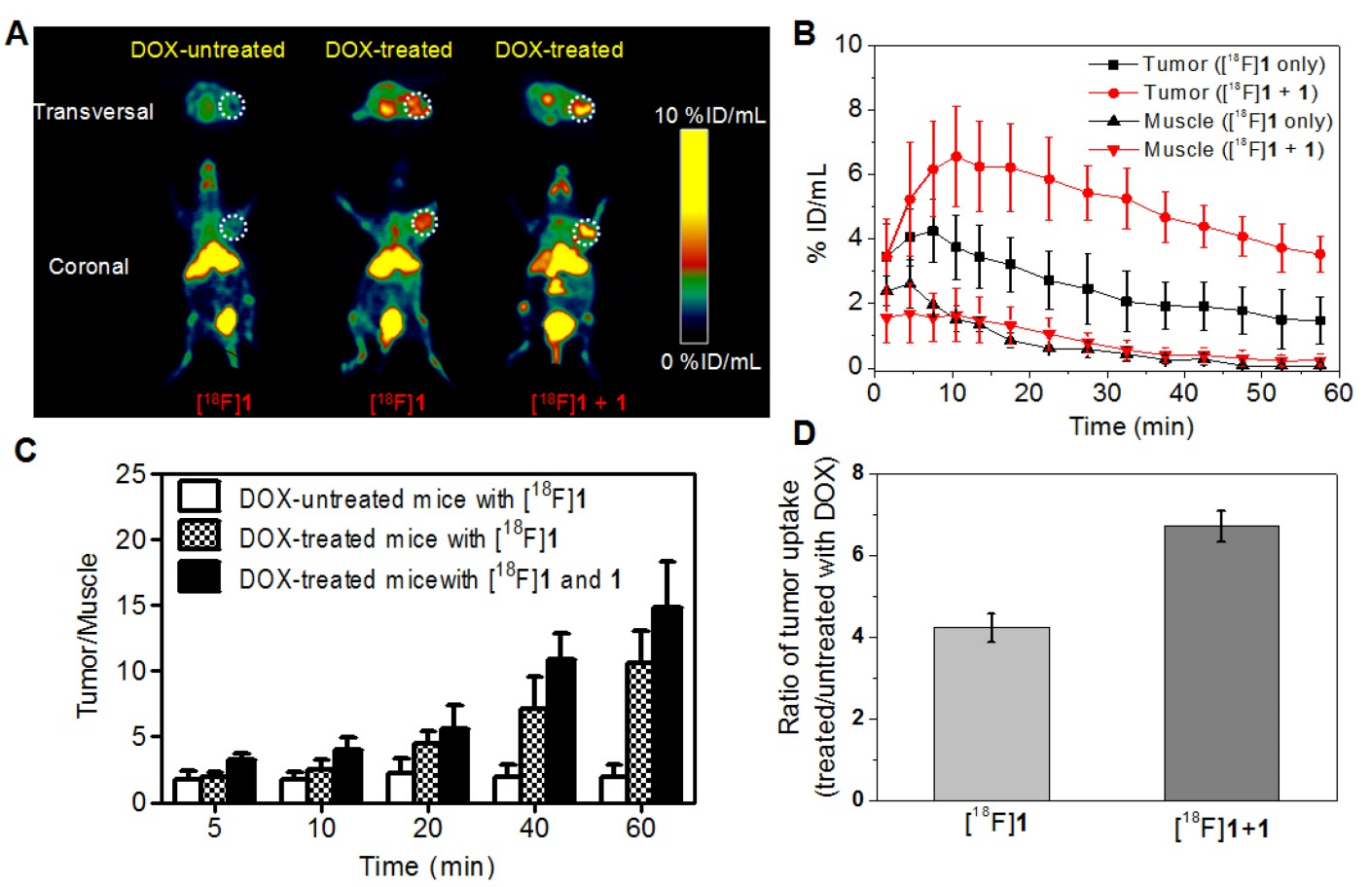
MicroPET imaging of mice bearing HeLa xenograft tumor before and after DOX treatment via intratumoral injection. (A) Transversal and coronal PET images at 15-20 min post injection. (B) Time course of radiotracer uptake and retention in tumor and muscle of DOX-treated mice. (C) Quantified tumor-to-muscle (T/M) uptake ratio. (D) Uptake ratio of radiotracer in DOX-treated versus untreated tumors at 15-20 min post injection. Error bars represent standard deviation (n = 3).

Figure 4A showed representative PET images of mice bearing HeLa xenograft tumor at 15-20 min post injection of [^18^F]**1** or [^18^F]**1** + **1** before and after DOX treatment. As expected, the DOX-treated group displayed obviously higher uptake of radiotracer in the tumor than the untreated group. Also noteworthy was that the DOX-treated tumor injected with only [^18^F]**1** could be clearly visualized at 15-20 min, while the co-injection of [^18^F]**1** and **1** produced much higher accumulation of radiotracer in the tumor and better tumor-to-background contrast for PET imaging in both transversal and coronal positions (Figure 4A). From the imaging results, one can also observe that the liver and kidney showed relatively high absolute uptake of radioactivity, suggesting that the radiotracer might clear through the hepatic and renal metabolism.

Quantification of PET images revealed that the tumor uptake (%ID/mL) in the DOX-treated group with injection of only [^18^F]**1** quickly reached 4.25 ± 0.97 %ID/mL at 7.5 min and then decreased gradually to 1.48 ± 0.73 %ID/mL at 57.5 min, whereas that in the DOX-treated group with co-injection of [^18^F]**1** and **1** showed higher radiotracer uptake of 6.57 ± 1.56 and 3.54 ± 1.56 %ID/mL at 10.5 and 57.5 min, respectively (Figure 4B). It is interesting to note that in both DOX-treated and untreated groups, the radioactivity in muscle tissues was relatively low (Figure 4B and Figure S24) in spite of injection with only [^18^F]**1** or co-injection with [^18^F]**1** and **1**. As a result, images with high signal-to-noise ratio could be observed especially for the DOX-treated group co-injected with [^18^F]**1** and **1**. As shown in Figure 4C, the T/M uptake ratio in the untreated group only increased from 1.74 to 2.18 during 60 min's microPET scanning, while that in the DOX-treated group with injection of only [^18^F]**1** increased significantly from 1.88 to 10.52 and the co-injection of [^18^F]**1** and **1** even led to the T/M increasing from 3.08 to 14.81. Furthermore, the tracer uptake ratio in DOX-treated versus untreated tumors at 15-20 min post injection of only [^18^F]**1** was 4.23, while the tracer uptake ratio for DOX-treated versus untreated tumors with co-injection of [^18^F]**1** and **1** was 6.72 (Figure 4D). These results not only demonstrated the high sensitivity and specificity of [^18^F]**1** for detection of tumor apoptosis, but also validated the advantage of co-injection strategy in improving the imaging effect.

**Figure 5:**
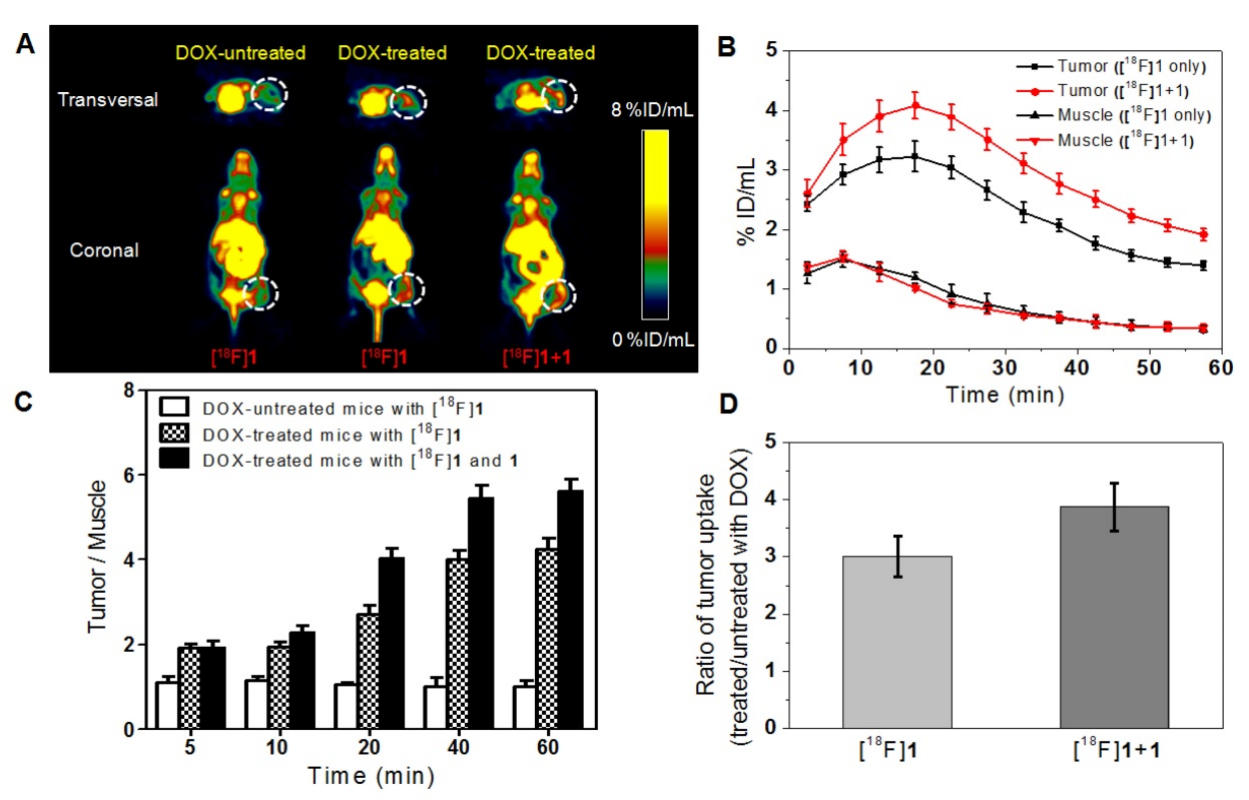
MicroPET imaging of mice bearing HeLa xenograft tumor before and after DOX treatment via intravenous injection. (A) Transversal and coronal PET images at 15-20 minpost injection. (B) Time course of radiotracer uptake and retention in tumor and muscle of DOX-treated mice. (C) Quantified uptake ratio of tumor to muscle (T/M). (D) Uptake ratio of radiotracer in DOX-treated versus untreated tumors at 15-20 min post injection. Error bars represent standard deviation (n = 3).

Considering the intratumoral injection is not standard for modeling clinical applications, we have also performed microPET imaging on the tumor-bearing mice with intravenous injection of DOX for comparison so as to study the effect of intravenous injection of DOX on the probe uptake in apoptotic and non-apoptotic tumors. As shown in Figure 5A, high peritoneal uptake was also observed because of the hepatic and renal clearance of the radiotracer as mentioned above and the DOX-treated group also displayed higher tumor uptake than the DOX-untreated group as anticipated. In the DOX-treated groups with injection of only [^18^F]**1** or co-injection of [^18^F]**1** and **1**, the activity uptake profile in tumors was in accordance with that in the intratumoral injection (DOX) group, and the highest tumor uptake was determined to be 3.22 ± 0.25 and 4.08 ± 0.22 %ID/mL at 15-20 min, respectively (Figure 5B). Similarly, the radioactivity in muscle tissues was also relatively low in both groups with injection of only [^18^F]**1** or co-injection of [^18^F]**1** and **1** (Figure 5B). The T/M uptake ratio in the DOX-treated group with injection of [^18^F]**1** was determined to be 4.24 at 60 min, while that in the DOX-treated group with co-injection of [^18^F]**1** and **1** was determined to be 5.62 at 60 min (Figure 5C), which decreased remarkably in comparison with those in the DOX-treated group via intratumoral injection (DOX, 0.2 mg). Moreover, compared with the intratumoral injection group, the tracer uptake ratio in DOX-treated versus untreated tumors via intravenous injection decreased slightly to 3.01 for the group injected with only [^18^F]**1** and 3.87 for that co-injected with [^18^F]**1** and **1**, respectively (Figure 5D). This might be due to that intravenous injection of DOX induced relatively lower expression level of active caspase-3 in the tumor than intratumoral injection of DOX. Although the tumor uptake ratio in the groups intravenously injected with DOX was slightly lower than that in the groups intratumorally injected with DOX, however, the probe [^18^F]**1** could still detect the tumor apoptosis induced by drugs. As a whole, all the results demonstrated that the probe [^18^F]**1** could be used to effectively evaluate the early response of tumor to the treatment and the co-injection strategy could enhance the signal retention and improve the imaging effect.

### Histological analysis

**Figure 6:**
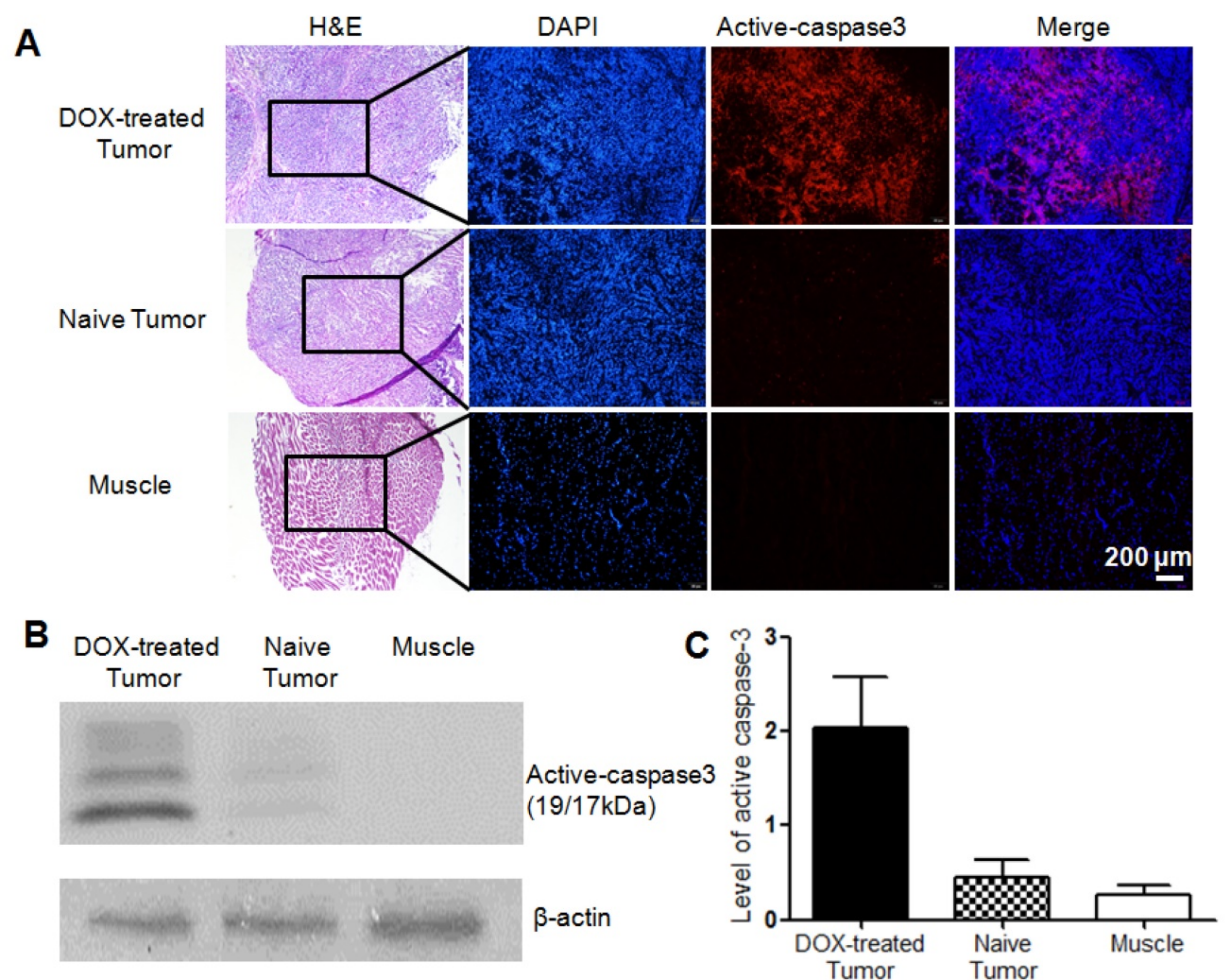
Histopathologic analysis of different tissues in mice bearing HeLa xenograft tumor. (A) H&E staining and immunofluorescence staining for DOX-treated tumor via intratumoral injection (top), naive tumor (middle) and muscle (bottom). Red fluorescence denoted the activated caspase-3 in DOX-treated tumor. Scale bar, 200 μm. (C) Activation of caspase-3 determined by western blotting analysis. (C) Quantification of activated caspase-3 band intensities normalized to the loading control β-actin. Error bars represent standard deviation (n = 3).

The level of activated caspase-3 in tumors with or without apoptosis induction was further analyzed and compared by histochemical staining and western blotting analysis. As can be observed from the histological staining (Figure 6A), H&E staining showed cells in the tumor or muscle tissues, and DAPI staining displayed cell nucleus with blue fluorescence, while red fluorescence demonstrated activated caspase-3 in apoptotic cells. From Figure 6A, no obvious red fluorescence was observed in the untreated tumor or muscle of the DOX-treated mice. On the contrary, obvious red fluorescence signal could be observed in both DOX-treated tumors via intratumoral or intravenous injection (Figure 6A and Figure S25A), although the former showed stronger red fluorescence than the latter. The overlay also confirmed that apoptosis occurred in the tumor was induced by chemotherapy, while the untreated tumor exhibited the baseline homeostatic apoptosis throughout the tumor progression. Tumor lysates were further analyzed for determining the expression level of activated caspase-3 (19/17 kDa). The western blotting results revealed that the level of activated caspase-3 increased distinctly regardless of comparing with the untreated tumor or the background of muscle (Figure 6B). Quantification of the band intensities normalized to the loading control β-actin showed that the expression level of activated caspase-3 in DOX-treated tumor was 4.5 and 7.5 folds higher than that in the untreated tumor and muscle, respectively (Figure 6C). Moreover, for the treated groups, the tumor with intravenous injection of DOX showed a slightly lower expression level of active caspase-3 than the intratumoral injection group (Figure S25B). This well explained the above microPET imaging results that the tumor uptake ratio in the group intravenously injected with DOX was slightly lower than that in the group intratumorally injected with DOX.

## Discussion

According to the strategy developed recently, the possible mechanism of action was proposed for the activatable apoptosis-specific imaging probe [^18^F]**1** and depicted in Scheme 1. At first, following cellular uptake of the probe [^18^F]**1**, the disulfide bond of cysteine can be cleaved in the reducing environment of the tumor cell. Subsequently, the drug-induced active caspase-3/7 will specifically trigger the activation of the peptide in the probe [^18^F]**1**, including cleaving the DEVD sequence and releasing the amino group of cysteine. Then the 1,2-aminothiol group undergoes a bioorthogonal condensation with the 2-cyano group of CBT to form a macrocyclized product (**1**-dimer) 21, and more rigid and hydrophobic oligomers will be self-assembled in situ due to intermolecular π-π stacking interactions, which will result in a high density of radioactive signal for sensitive and specific detection of the effector enzyme in cells or living animals. This strategy combines the activatable probe in imaging the caspase-3 activity with amplified signal, and has been successfully realized in the fluorescence imaging of active caspase-3 in apoptotic cells 30, magnetic resonance imaging 31,32 and PET imaging 21-23 of tumor apoptosis in mice. These probes provided sensitive techniques for monitoring the subcellular effect of applied therapy prior to the change of tumor size. In the present study, the caspase-triggered signal amplification strategy combined with a rapid and efficient [^18^F] radiolabelling approach was applied to develop a novel stable PET tracer.

The study began with the synthesis of non-radioactive compound **1** (namely the cold precursor) and the target probe [^18^F]**1**. The chemical synthesis of **1** was facile and straightforward, and high yield was obtained after purification. It was found that the precursor **1** was very stable in pyridazine-HCl buffer even at the high temperature of 100 °C, indicating that the [^18^F]fluorination process could be conducted in a broad temperature range. In addition, the probe **1** at a high concentration exhibited non-toxicity to normal cells and tumor cells, indicating it had good biocompatibility. The probe could be activated by the cleaved caspase-3 enzyme to undergo an intermolecular macrocyclization and nanoaggregation, which firmly verified the established mechanism of caspase-3/7-triggered signal amplification for effectively imaging the activity of caspase-3/7 21. These preliminary results are very encouraging for the *in vivo* biological evaluation of the novel probe.

As for the preparation of the target probe [^18^F]**1**, a desirable [^18^F]fluorination approach has been applied, where both drying of [^18^F]fluoride ion and HPLC purification can be obviated. The tracer could be obtained in a simple and rapid kit-like manner with high RLY and RCP. [^18^F]C-SNAT, the recently reported caspase-sensitive nanoaggregation tracer, was radiosynthesized with an overall RLY of 3.2 ± 0.1% and the specific activity of 63 ± 7.4 MBq/nmol, and the whole process took 3-3.5 hours 21. Even after the radiolabelling conditions were optimized, the radiosynthesis of [^18^F]C-SNAT still took 3 hours, with the overall RLY of 14.4 ± 0.4 % and specific activity of 101.8 ± 31.7 MBq/nmol 22. However, based on our established labeling approach, the whole radiosynthesis process could save at least 2.5 hours and the RLY of [^18^F]**1** could be improved by about four folds with a similar activity concentration. More importantly, the stability of the probe [^18^F]**1** in serum was significantly improved since it was very stable (> 97%) within 4 h in mouse serum at 37 °C, while only 60% of intact [^18^F]C-SNAT could be observed after 1 h incubation in the mouse serum 21. The radiolabelling process was accomplished within a fully shielded hot cell and can be easily adapted to a simple synthesis module as well as a microfluidic reactor in a small space. Moreover, neither toxic metals nor noxious solvents were used during the labeling process. Therefore, the novel probe [^18^F]**1** can be produced with a simple, rapid, safe and economical methodology, which will be very useful for large-scale production and clinical applications.

Considering the probe [^18^F]**1** is easily soluble in saline and very stable in mouse serum, it is beneficial for transportation and further biological studies. Compared with either [^18^F]FDG or [^18^F]ML-10 23 with no differentiated response to cell death, the tracer [^18^F]**1** showed higher uptake in apoptotic cells in relation to drug-induced cell death in the tumor. In addition, the experimental results about [^18^F]**1** were also comparable to the previously reported [^18^F]C-SNAT with a similar mechanism of action, demonstrating that the activation of caspase-3 can trigger these probes in living cells and enhance the retention of the radioactivity 21. Accordingly, the efficient radiosynthesis method along with good *in vitro* properties encouraged us to verify the practical value of the probe [^18^F]**1** for *in vivo* imaging.

As expected, the radiotracer [^18^F]**1** displayed a high tumor uptake and a high tumor-to-muscle uptake ratio in the mice with subcutaneously xenografted HeLa tumors after the treatment of DOX. As shown in Figure 4 and Figure 5, microPET imaging of apoptotic tumors with the co-injection of [^18^F]**1** and **1** was obviously better than that with the injection of [^18^F]**1** alone, suggesting that the cold compound **1** could promote the caspase-controlled intracellular condensation and self-assembly of [^18^F]**1** into nanoparticles (i.e., [^18^F]**1**-NPs) in tumors 34. Based on the proposed mechanism, microPET imaging with the co-injection of [^18^F]**1** with **1** and the injection of [^18^F]**1** alone both showed an increasing trend in the tumor uptake over time regardless of intravenous or intratumoral administration of DOX, and thus the difference between the treated apoptotic tumor and the untreated tumor became more and more obvious. Although the radiotracer uptake in the tumor with intravenous injection of DOX was slightly lower than that in the group with intratumoral injection of DOX, which resulted from the relatively lower expression level of activated caspase-3 in the former group, the tracer uptake ratio in DOX-treated versus untreated tumors was still at an acceptable level. The tumor-to-muscle uptake ratio in the treated apoptotic tumor model was more than two folds of that in the untreated group at 20 min post injection, which correlated well with the level of drug-induced apoptosis. This early readout of treatment response showed that our designed probe could be used as an accurate predictor of therapy outcome.

Recently, a potential PET imaging agent [^18^F]ICMT-11 for monitoring the tumor cell death has been approved for clinical trials, where only a modest increase of 65% uptake can be observed in the treated tumor in comparison with the vehicle control tumor 36. Similarly, little differential response was observed for other established radiotracers, such as ^18^F-FDG, ^99m^Tc-Annexin V, and ^18^F-ML-10 23. However, our PET imaging results demonstrated that probe [^18^F]**1** can efficiently image the caspase-3 activity in drug-treated tumors *in vivo* and both the caspase-3 activation and the intermolecular cyclization were essential for the enhanced imaging contrast in apoptotic tumors. As a whole, the currently developed probe [^18^F]**1** could provide an early readout of therapeutic efficacy and allow the selection of the most appropriate treatment method for achieving optimal clinical efficacy.

## Conclusions

A novel caspase-responsive PET tracer [^18^F]**1** was designed and successfully radiosynthesized with a simple, rapid, safe and economical methodology. *In vitro* and *in vivo* biological evaluations indicated that [^18^F]**1** possessed good stability, favorable pharmacokinetics and high signal-to-noise ratio in imaging the therapeutic efficacy of DOX on the cervical cancer, and the co-injection strategy of [^18^F]**1** and **1** led to significantly increased tracer uptake in the tumor and remarkably enhanced signal-to-noise ratio, which correlated well with the expression level of active caspase-3 in drug-induced apoptotic tumor. The early readout of treatment response ensures that the tracer can serve as a promising apoptosis-specific PET tracer for real-time monitoring the tumor response to the therapy. The clinical value of probe [^18^F]**1** in evaluating the chemotherapeutic or radiotherapeutic effect on other tumors is underway.

## Supplementary Material

Supplementary figures and tables.

## Abbreviations

PET: positron emission tomography
DEVD: Asp-Glu-Val-Asp
RLY: radiolabelling yield
RCP: radiochemical purity
log *P*: octanol/water partition coefficient
DOX: doxorubicin
T/M: tumor to muscle
MRI: magnetic resonance imaging
CT: computed tomography
PEG: polyethyleneglycol
CBT: 2-cyanobenzothiazole
ROI: region-of-interest
DAPI: 4',6-diamidino-2-phenylindole
[^18^F]C-SNAT: caspase-sensitive nanoaggregation tracer
HPLC: high performance liquid chromatography
MS: mass spectrometry
TEM: transmission electron microscope
DLS: dynamic light scattering
DMF: N,N-dimethylformamide
