# Supplementary material for: Rational design of caspase-responsive smart molecular probe for positron emission tomography imaging of drug-induced apoptosis
Source: Theranostics. 2019 Sep 21;9(23):6962–75. doi: 10.7150/thno.35084 (PMC6815954; doi:10.7150/thno.35084)
Supplement: Supplementary file 1 — Supplementary figures and tables. [file thnov09p6962s1.pdf]

Supplementary Material

Rational design of caspase-responsive smart molecular probe for positron emission tomography imaging of drug-induced apoptosis

Ling Qiu[1], Wei Wang[1], Ke Li[1], Ying Peng[1], Gaochao Lv[1], Qingzhu Liu[1], Feng Gao[2,*], Yann Seimbille[3], Minhao Xie[1,*], Jianguo Lin[1,*]

1. Key Laboratory of Nuclear Medicine of Ministry of Health, Jiangsu Key Laboratory of Molecular Nuclear Medicine, Jiangsu Institute of Nuclear Medicine, Wuxi, Jiangsu, China
2. Department of Medical Imaging, Jinling Hospital, Medical School of Nanjing University, Nanjing, Jiangsu, China
3. Department of Radiology and Nuclear Medicine, University Medical Center Rotterdam, Erasmus MC, Wytemaweg 80, 3015 CN Rotterdam, The Netherlands

*Corresponding authors: Jianguo Lin, Ph.D., E-mail: linjianguo@jsinm.org; Minhao Xie, Ph.D., E-mail: xieminhao@jsinm.org; Feng Gao, Ph.D., E-mail: rggaofeng@hotmail.com.

General method

All chemicals from commercial sources were of reagent grade and used without further purification. Phosphate buffered saline (PBS, 0.01 M, pH 7.4) was purchased from Sangon Biotech Co. Ltd. (China). The reagent 3-[4,5-dimethyl-2-thiazolyl]-2,5-diphenyl-2-tetrazolium bromide (MTT) used for cytotoxicity study was purchased from Sigma (USA). Dulbecco's modified eagle medium (DMEM) and RPMI 1640 were purchased from Gibco (USA). Human cervical cancer cell line HeLa, human normal gastric mucosal epithelial cell line GES-1, human normal liver cell line LO2 and human normal renal epithelial cell line HEK293 were obtained from the Cell Bank of Chinese Academy of Sciences (China). BALB/c nude mice (18-20 g, 4-6 weeks old) were purchased from SLAC Laboratory Animal Co. Ltd. China) for animal experiments. Radioimmunoprecipitation assay (RIPA) lysis buffer and antibody against active caspase-3 were purchased from Beyotime (Beyotime Institute of Biotechnology, China). Antibody against β-actin, HRP-conjugated secondary antibodies and western blotting luminol reagent were purchased from Santa Cruz Biotechnology, Inc. (USA). All procedures involving animals were approved by the Animal Care and Ethnics Committee of Jiangsu Institute of Nuclear Medicine.

Electron spray ion mass spectra (ESI-MS) were determined using a Waters Platform ZMD4000 LC/MS (Waters, USA). Nuclear magnetic resonance spectrometer (^{1}H-NMR, ^{13}C-NMR and ^{19}F-NMR, Bruker DRX-400; Bruker; Germany) was used to obtain the spectra of samples dissolved in d_6-DMSO, and the chemical shift was referenced to tetramethylsilane (TMS). The high performance liquid chromatography (HPLC) system was equipped with a pump (Waters 1525 HPLC; Waters; USA), and connected to reverse phase column (RP-C18; 4.6 × 250 mm; 10 um; Elite Analytical Instrument Company; China), a UV detector (2487 dual wavelength absorbance; Waters; USA) as well as a radioactivity detector (Radiomatic 610TR; Perkin Elmer; USA) which were operated by software programs Breeze (NY; USA) and proFSA (Perkin Elmer; USA). The compounds were analyzed with a gradient analysis method (Method A, Table S1). TEM samples were prepared by dispersing the particles onto holey carbon films on copper grids. The micrographs were carried out on Hitachi (H-7650) operated at an acceleration voltage of 80 kV. The zeta potential and size distribution of the nanoparticles were analyzed on a Nano-ZS instrument (Malvern Instruments Limited). The activity was counted using a γ counter (Packard-multi-prias; Perkin Elmer; USA). Micro-PET

imaging was performed on an Inveon scanner (Siemens, Germany).

Statistical analysis

Results were expressed as mean ± standard deviation (SD) unless otherwise stated. Statistical comparison between two groups was determined by t-test, and that between three or more groups was analyzed by one-way ANOVA followed with a post-hoc Tukey's HSD test. Time course analysis between groups was performed by general linear model repeated-measures analysis. Correlation analyses were performed by one-tailed Spearman r (non-parametric). For all tests, $p < 0.05$ was considered statistically significant. All statistical calculations were performed using GraphPad Prism v.5 (GraphPad Software Inc., USA), except for general linear model analyses that were performed using SPSS (IBM).

Chemical synthesis and characterization of probe 1

The chemical synthesis of cold probe **1** was performed according the method as shown in the following Figure S1.

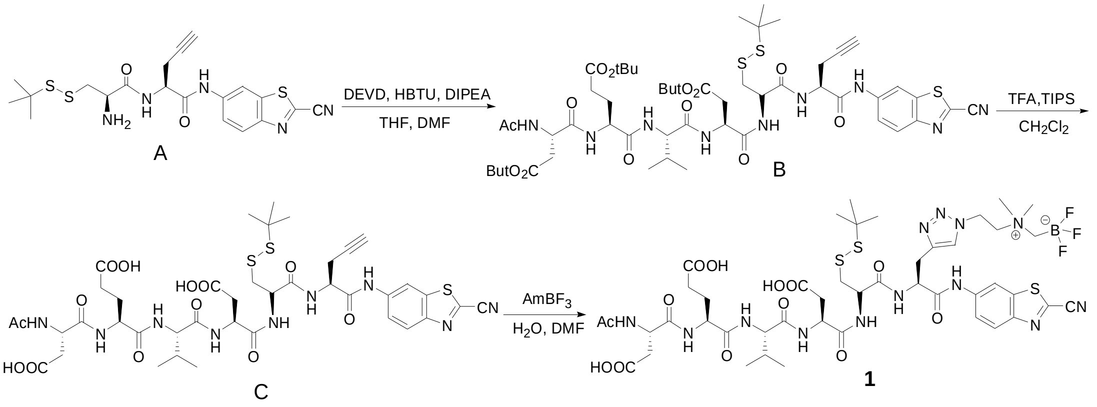

Figure S1. Synthesis of the precursor **1**.

Synthesis of the compound B

The compound **A** and peptide sequence DEVD were synthesized according to the method published previously.[1,2] To a solution of compound **A** (150 mg, 0.325 mmol, 1eq) in dry THF (4 mL),protected Ac-DEVD (246 mg, 0.38 mmol, 1.1 eq), HBTU (142 mg, 1.15eq) and DIPEA (142 μL, 2.5 eq)were added. The resulting solution was stirred for 2 hours at room temperature and then evaporated under reduced pressure. The crude product was

purified by preparative HPLC (Method B, Table S2) to obtain the compound **B** (300 mg, 82 %).

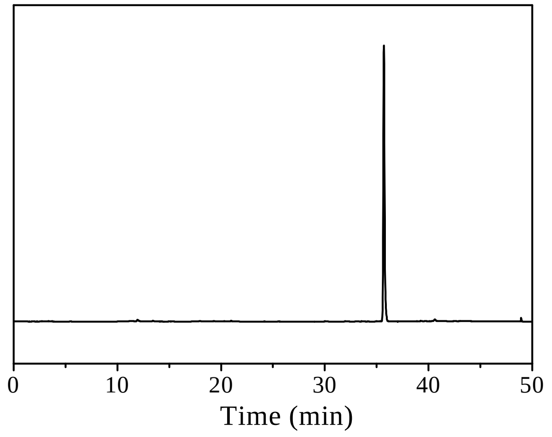

Figure S2. HPLC trace of purified **B**.

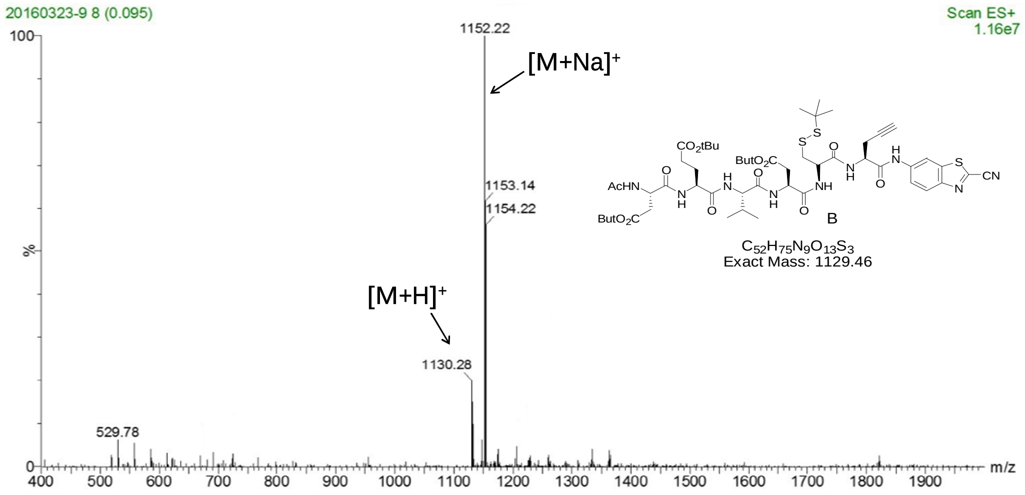

Figure S3. ESI-MS of compound **B**.

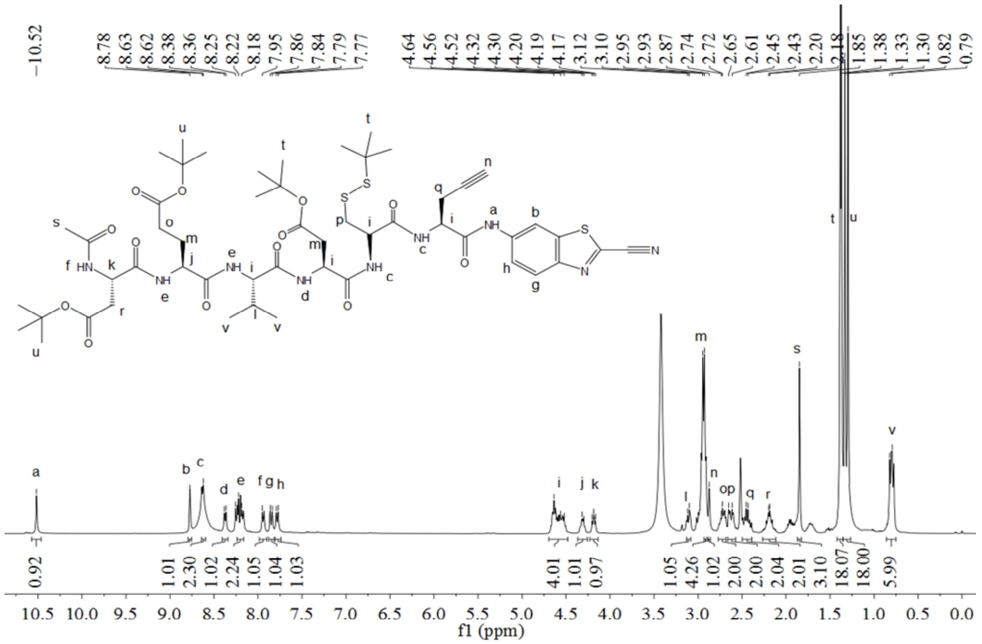

Figure S4. ^{1}H NMR of compound **B**.

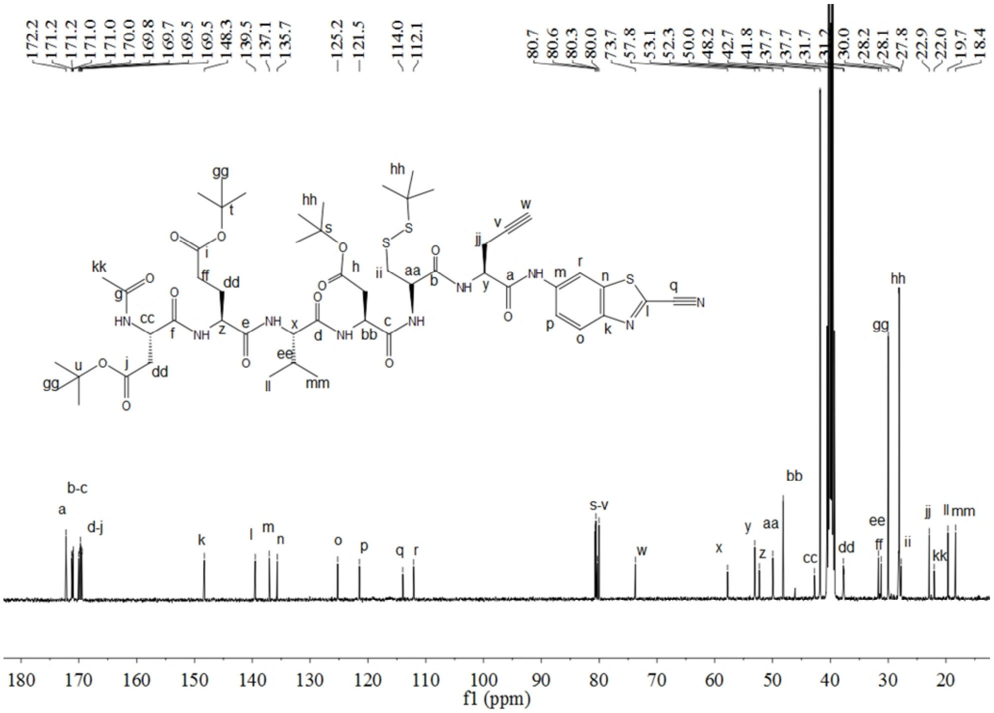

Figure S5. ^{13}C NMR of compound **B**.

Synthesis of the compound C

To remove the protecting group t-Bu, TFA (3 mL) and TIPS (60 μL) were added to a solution of compound **B** (300 mg, 0.265 mmol) in DCM (3 mL) and stirred at room

temperature for 1 h. Then the solvent was evaporated under reduced pressure and the crude product was precipitated from the cleavage solution with cold diethyl ether to remove the protecting groups. The crude product **C** (282 mg) was used without further purification.

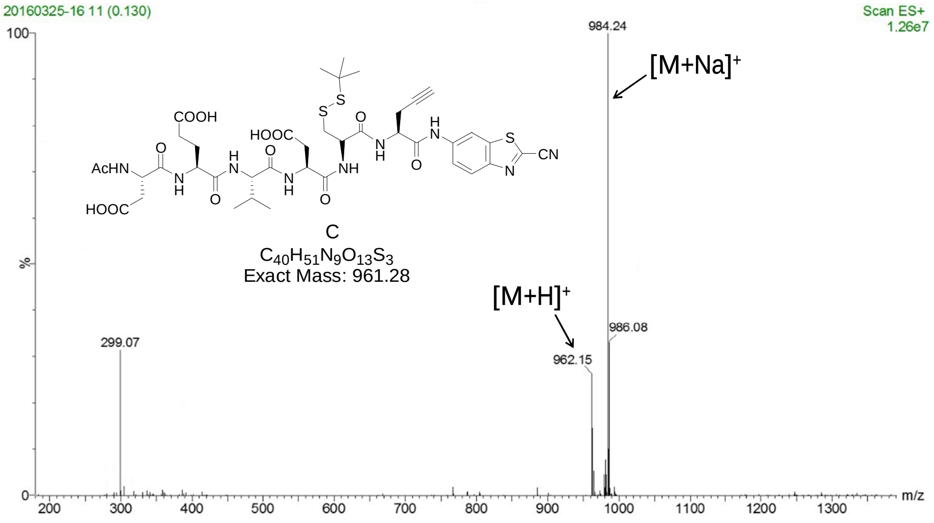

Figure S6. ESI-MS of compound **C**.

Synthesis of the compound 1

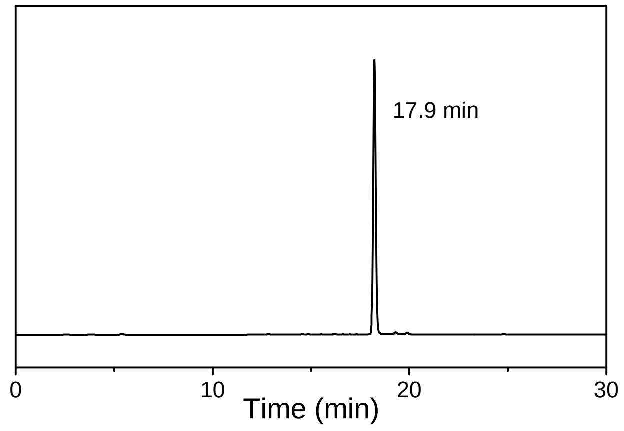

Figure S7. HPLC trace of purified **1**.

The intermediate 2-azidoethyl-N,N-dimethylammoniomethyltrifluoroborate ($AmBF_3$) was obtained according to the method reported previously.[3] To a solution of compound **C** (115 mg, 1 eq) in N,N-Dimethylformamide (DMF) (5 mL) was added $AmBF_3$ (36 mg, 1.5 eq). After tris(2-benzimidazolylmethyl)amine $(BimH)_3$ (4.8 mg, 0.1 eq) was added to the solution, sodium ascorbate (48 mg, 2 eq) and $CuSO_4$ (6 mg, 0.2 eq) in water and HEPES (0.2 M, 1.0 mL) were added. The resulting solution was heated to 45 °C for 2 hours. The crude product

was purified by preparative HPLC (Method C, Table S3) to obtain the compound **1** (138 mg, 72 %).

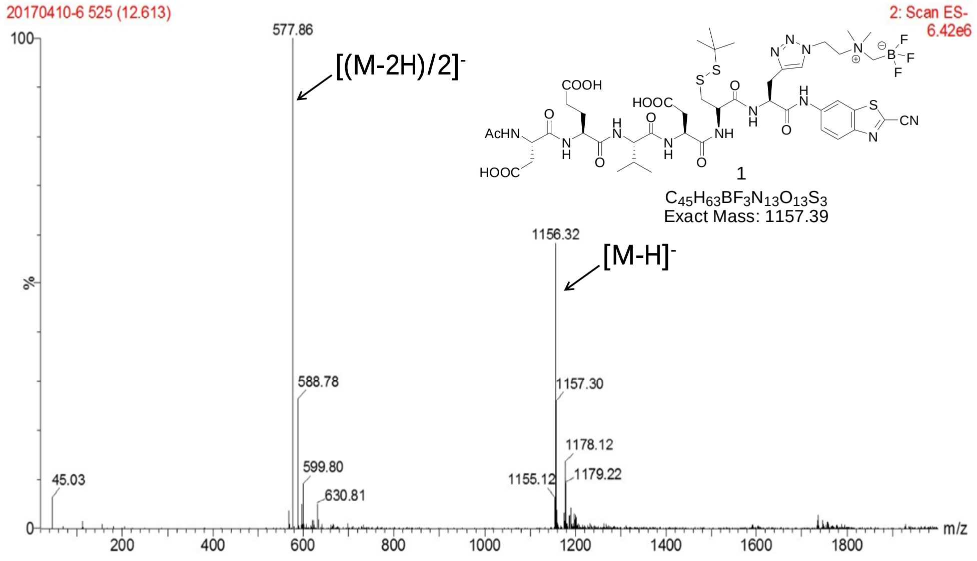

Figure S8. ESI-MS of compound **1**.

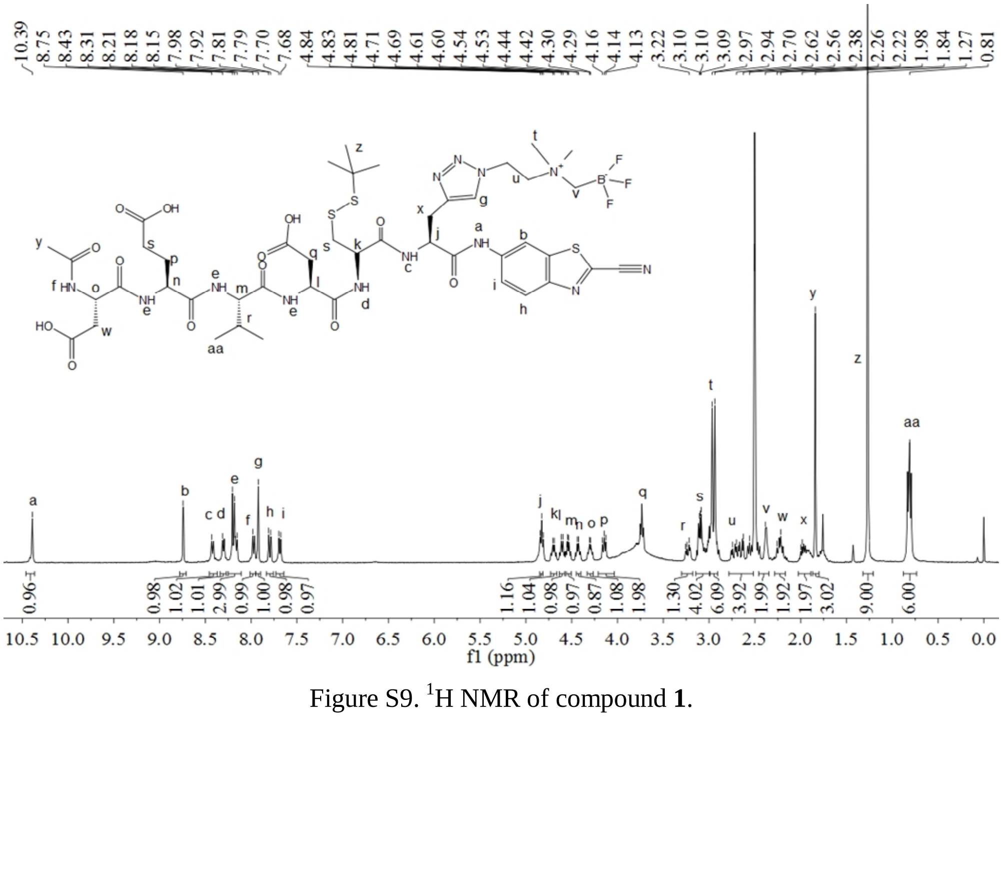

Figure S9. ^{1}H NMR of compound **1**.

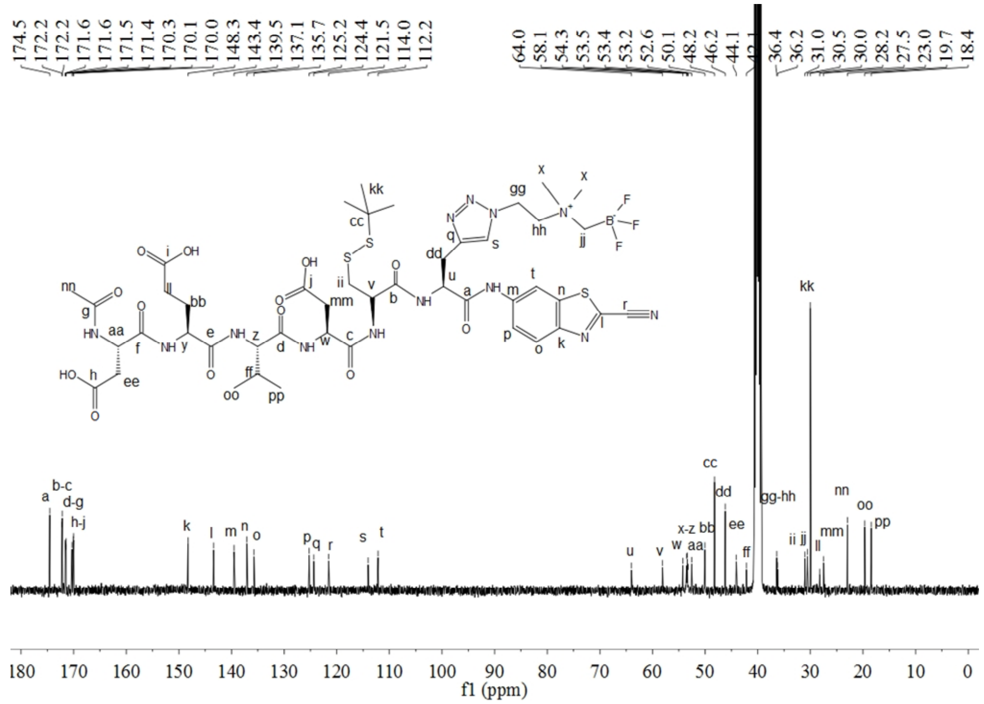

Figure S10. ^{13}C NMR of compound **1**.

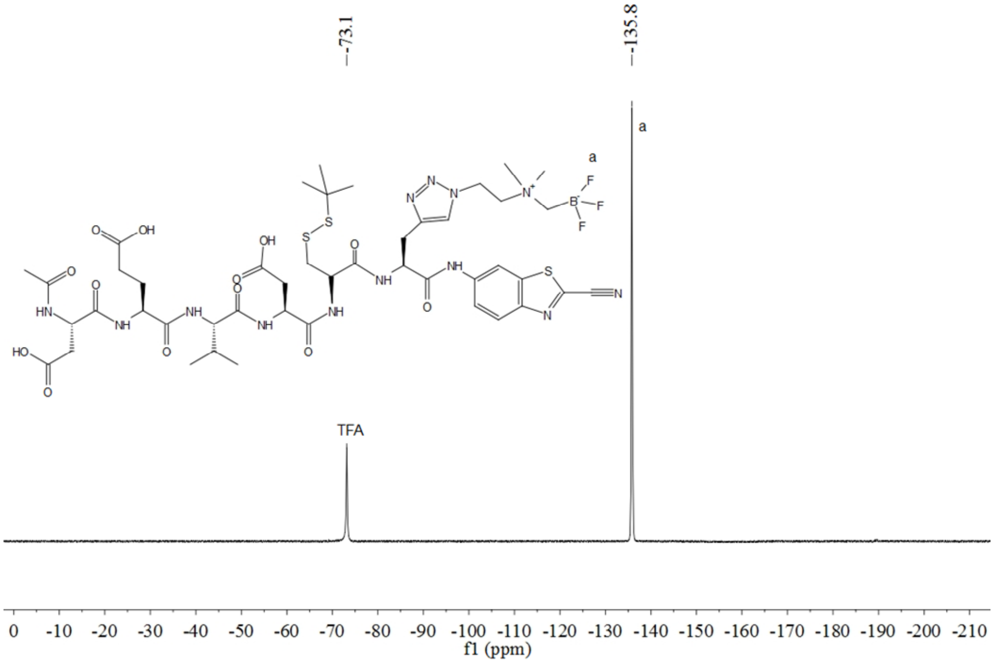

Figure S11. ^{19}F NMR of compound **1**.

Synthesis of the probe 1-FITC

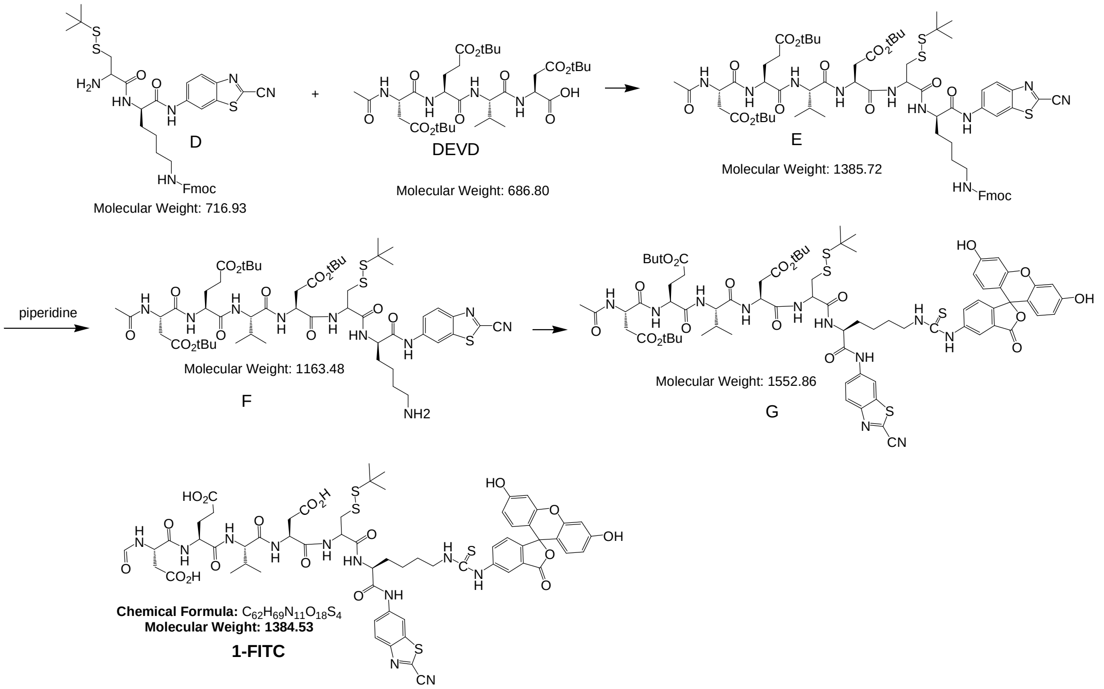

Figure S12. Synthesis of the probe **1-FITC**.

Compound **D** was synthesized according to our previous report[1], except for using Boc-Lys(Fmoc)-OH instead of N-Boc-propargylglycine. Then compound **E** was synthesized by conjugation of DEVD with compound **D**. The Fmoc group in compound **E** was deprotected using 20% piperdine to obtain compound **F**. Compound **G** was obtained by reaction of compound **F** with fluorescein isothiocyanate isomer I (NCS-FITC) in the present of DIPEA. Compound **G** was treated with TFA and purified withby preparative HPLC to obtain **1-FITC**. The purity of probe **1-FITC** was checked by analyticalHPLC and the structure was confirmed using ESI-MS. ESI-MS: m/z $[M+H]^+$ calcd for $C_{62}H_{70}N_{11}O_{18}S_4$: 1384.5; found: 1384.2.

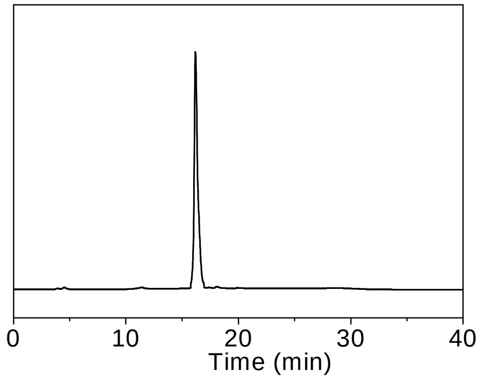

Figure S13. HPLC trace of probe **1-FITC**.

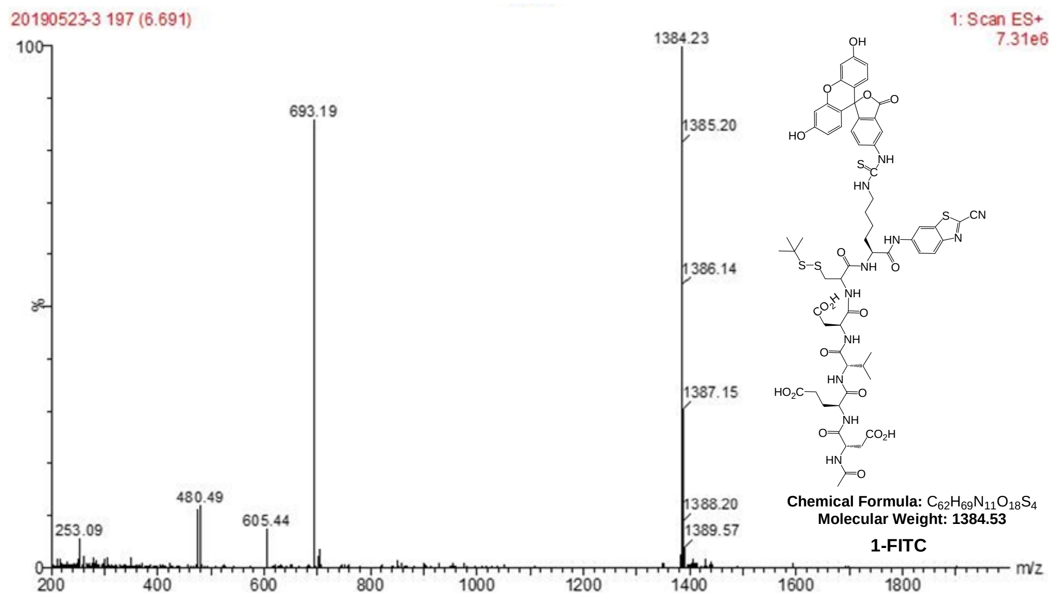

Figure S14. ESI-MS of probe **1-FITC**.

Stability of 1 in pyridazine-HCl buffer

The stability of cold probe **1** in pyridazine-HCl buffer (1.0 M, pH 2.0-2.5) was studied at different temperature. Briefly, **1** was dissolved in pyridazine-HCl buffer and then incubated at various temperature (50, 60, 70, 80, 90 and 100 °C) for 20 min, respectively. Afterwards, the sample was taken out for stability determination with analytical HPLC using Method A (Table S1).

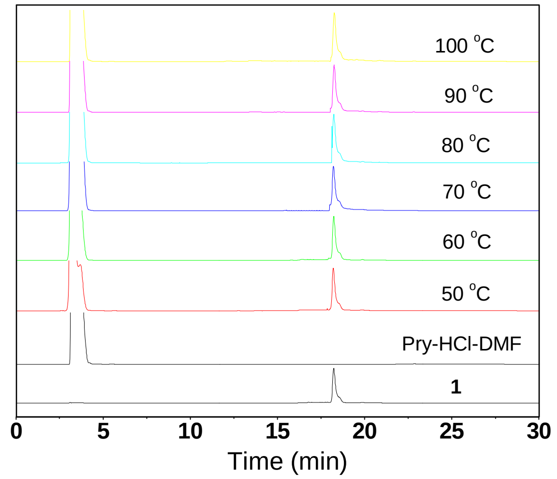

Figure S15. Stability assay of **1** in pyridazine-HCl buffer (pH = 2.5) at different temperature.

In vitro characterization of reduction and macrocyclization of probe 1

To evaluate the reduction of disulfide in probe **1**, TCEP·HCl (66 μL, 50 mM) was added

to the PBS solution of **1** (100 μL, 3.3 mM) at pH 7.4. The mixture was fixed to 330 μL with the buffer and incubated for 1 h at room temperature. Another reaction for caspase-3 (4.9 × 10^{-3} U, 7.35 × 10^{-4} mg, Sigma) and probe **1** (25 μM, 1 mL) was performed in caspase buffer with HEPES (50 mM), NaCl (100 mM), EDTA (1 mM), TCEP (10 mM), 10% glycerol, and 0.1% CHAPS at pH 7.4. Reactions were carried out at 37 °C for 6 h. The mixture of both reactions was analyzed by HPLC and LC-MS, respectively. The morphology of probe **1** was studied by TEM and DLS analysis after reduction by TCEP·HCl (50 mM) and enzyme digestion by caspase-3 (4.9 × 10^{-3} U, 7.35 × 10^{-4} mg) for 6 h.

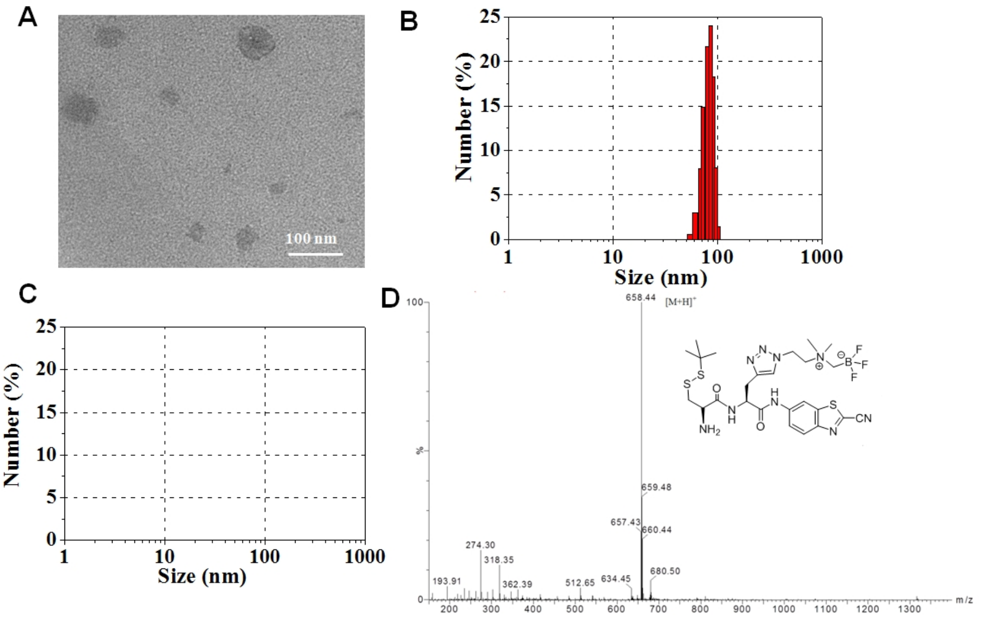

Figure S16. (A) TEM and (B) DLS analysis of probe **1** (25 μM) upon incubation with TCEP·HCl (50 mM) and caspase-3 (4.9 × 10^{-3} U, 7.35 × 10^{-4} mg, Sigma) for 6 h. (C) DLS analysis of probe **1** (25 μM) upon incubation with only caspase-3 (4.9 × 10^{-3} U, 7.35 × 10^{-4} mg, Sigma) for 6 h. (D) ESI-MS of reaction products of probe **1** only in the caspase-3 system.

Dimer formation in apoptotic HeLa cells

To further prove the formation of **1**-dimer in apoptotic tumor cells, **1** (100 μL, 3.3 mM) was added to the cell lysate of apoptotic HeLa cells induced by doxorubicin (DOX), and then incubated at 37 °C for 0 to 8 h. At the specific time point, the reaction mixture was analyzed by HPLC and the content of **1-dimer** was calculated according to HPLC analysis.

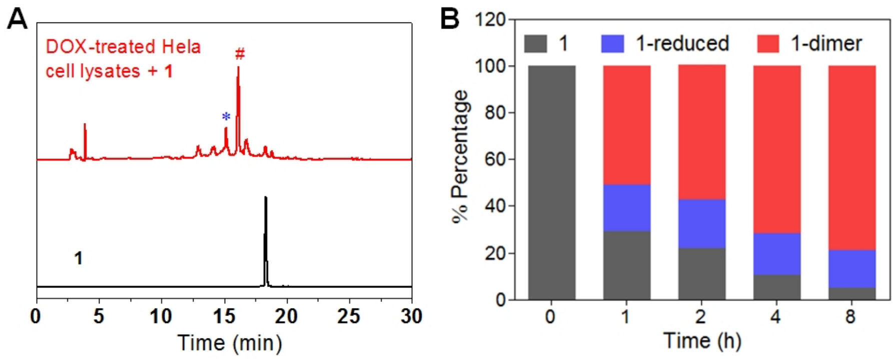

Figure S17. (A) HPLC traces of probe **1** incubated with the cell lysates of apoptotic HeLa cells for 8 h. Bottom: probe **1** alone; Top: reduced product (*) and cyclized product (#). (B) The content of **1-dimer** after incubation with the cell lysates of apoptotic HeLa cells at different time.

Enzyme kinetics study

For the enzyme-controlled cleavage kinetics studies, the probe **1** at different concentrations (0.1, 0.25, 0.5, 1, and 2 mM) was incubated with the caspase-3 (0.4 μg/1 mL) assay buffer (pH = 7.4) containing 50 mM HEPES, 100 mM NaCl, 1 mM EDTA, 1 mM DTT, 10% glycerol, and 0.1% CHAPS at 37 °C for 1 h, respectively. Then the caspase-3-cleaved intermediates were characterized by HPLC, and the kinetic parameters were determined according to the method reported previously.[4,5]

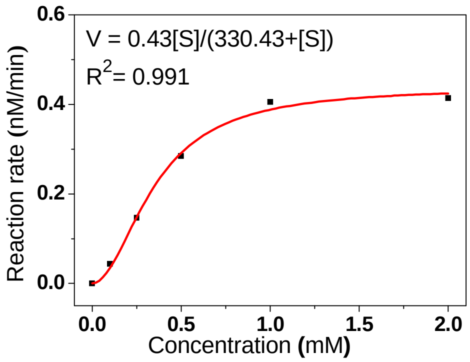

Figure S18. Kinetic studies for the cleavage of probe **1** towards caspase-3.

Biocompatibility of 1 and 1-NPs

One cancer cell line (HeLa) and three normal cell lines (GES-1, LO2 and HEK293) were cultured in DMEM supplemented with 10% fetal bovine serum (FBS, Gibco). The cells were grown in tissue culture dishes and kept in a humidified atmosphere of 5% CO_2 at 37 °C. The

medium was changed every other day. The cytotoxicity of **1** and **1-NPs** against HeLa, GES-1, LO2 and HEK293 cells was assessed by the traditional MTT assay. Firstly, 5 × 10^3 suspended cells per well were seeded into 96-well culture plate. The cells were allowed to grow for 12 h at 37 °C under 5% CO_2. Then **1** was dissolved in 0.1% DMSO and diluted with DMEM to the indicated concentration of 0, 6.125, 12.5, 25, 50 and 100 μM, respectively. The cells were exposed to various concentration of probe **1** and then cultured at 37 °C, with 5% CO_2 for 3, 6, 12, and 24 h, respectively. Then cells were treated with MTT (5 mg/mL, 20 μL) and incubated for another 4 h. For MTT assay, DMSO (150 μL) was added to each well when the medium was removed. The optical density (OD) of each well was determined at 470 nm using an ELISA reader. All tests were carried out in 6 repeats for at least three independent experiments. Cell viability% = (mean OD value of experimental group/mean OD value of control group) × 100%.

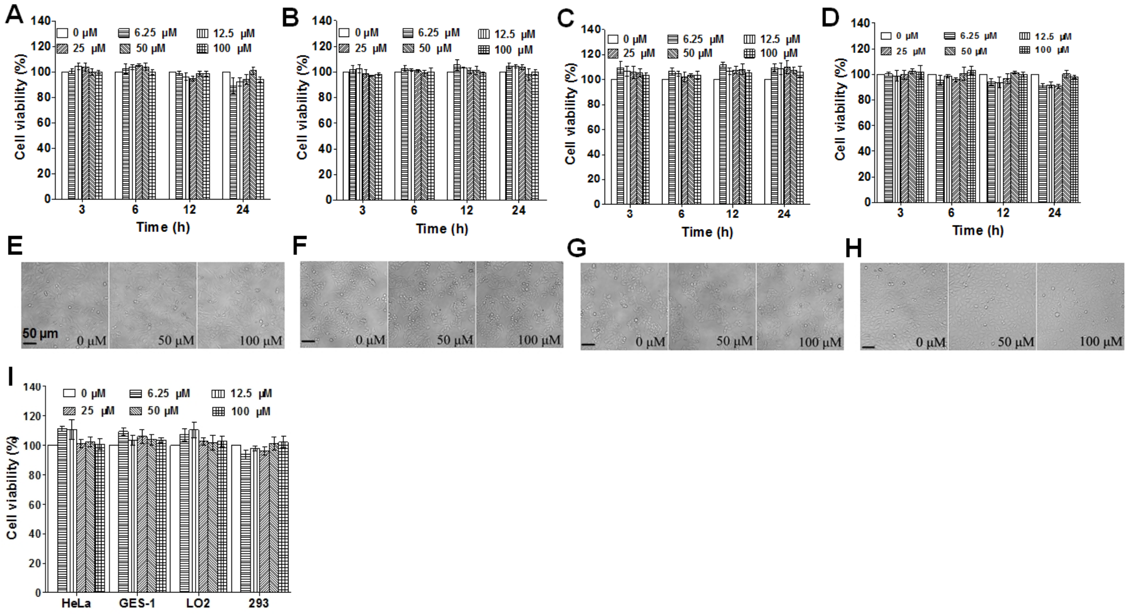

Figure S19. Cytotoxicity of **1** and **1-NPs** against cancer cell lines (HeLa) and normal cell lines (GES-1, LO2 and HEK293). Cell viability and morphology of HeLa (A, E), GES-1 (B, F), LO2 (C, G) and HEK293 (D, H) cells treated with **1** of different concentrations (0, 6.25, 12.5, 25, 50, and 100 μM) for 3, 6, 12 and 24 h, respectively. (I) Cytotoxicity of **1-NPs** against HeLa, GES-1, LO2 and HEK293 cell lines treated for 24 h. Error bars represent standard deviation (n = 3).

Radiolabelling and quality control of probe [^{18}F]1

For radiolabelling, a wet NCA (no carrier added) solution of [^{18}F]fluoride ion was used directly following trapping. The small QMA column efficiently traps Curie levels of NCA [^{18}F]fluoride ion, which are pre-conditioned with $NaHCO_3$ (10 mL, 0.5 mol/L) and deionized water (10 mL), respectively. The [^{18}F]fluoride ion was eluted with pyridazine-HCl buffer (400-1000 μL, pH = 2.0-2.5). The reaction tube containing [^{18}F]fluoride (200-500 mCi) and precursor **1** in DMF (5-20 μL, 25 nmol/μL) was heated at 80 °C for 20 min. Then the mixture was diluted and loaded onto a C_{18} light Sep-Pak cartridge, and free ^{18}F-fluoride was removed by flushing thrice with deionized water (10 mL). The desired probe [^{18}F]**1** was then eluted off the column with ethanol (0.5 mL), and diluted with more than 4.5 mL saline. Finally, the product was passed through a 0.22-μm sterile filter for further use. A small volume of sample was taken out for quality control by radio-HPLC.

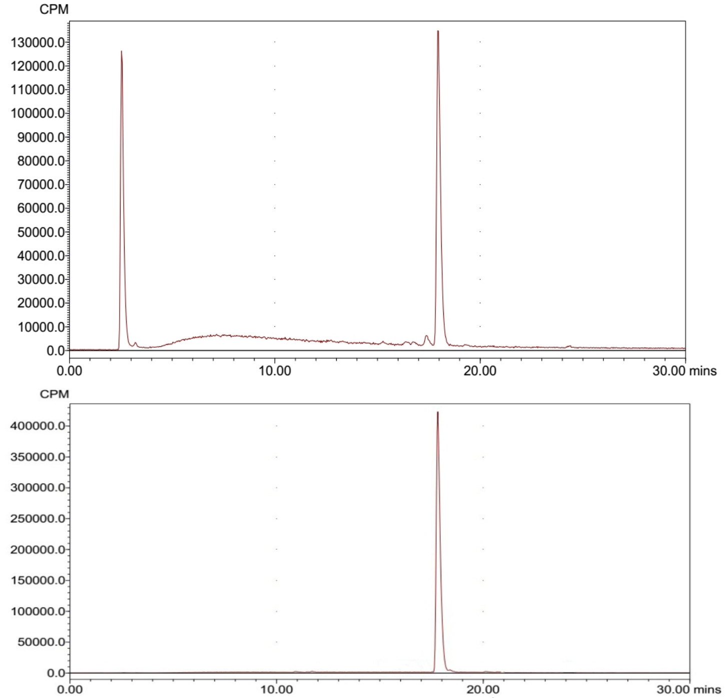

Figure S20. Radio-HPLC traces of the reaction mixture of [^{18}F]**1** (top) and purified [^{18}F]**1** (bottom), respectively.

Determination of partition coefficient (log *P*)

The octanol/water partition coefficient of probe [^{18}F]**1** was determined at pH = 7.0 by measuring the distribution of the radiotracer in n-octanol and PBS. Briefly, n-octanol (500 μL) was added into a polypropylene tube which was filled with 500μL of radiotracerin PBS (pH =

7.0). The mixture was oscillated on a vortex mixer at 25 °C for 20min. Then the mixture was centrifuged (4,000 g, 5 min) and then two layers were separated. The activity of samples (100 μL) in each layer was measured with a γ counter. The partition coefficient (log *P*) was calculated using the equation of log P = log (C_o/C_w), where C_o and C_w represented the activity of the radiotracer in n-octanol layer and water layer, respectively. The result was expressed as mean ± SD of the data from at least three independent experiments.

In vitro stability assay

The target probe [^{18}F]**1** (100 μCi, 3.7 MBq, 100 μL) was added to PBS (400 μL) and incubated at 37 °C for 0.5, 1, 2, and 4 h, respectively. At each time point, the mixture was diluted with acetonitrile (500 μL) and then analyzed by radio-HPLC. Similarly, the stability of [^{18}F]**1**in the mouse serum was also studied by incubating the probe [^{18}F]**1** (100 μCi, 3.7 MBq, 100 μL) with mouse serum (400 μL) at 37 °C for 0.5, 1, 2, and 4 h, respectively. At each time point, the mixture was precipitated with acetonitrile (500 μL) and centrifuged at 12,000 rpm for 5 min. Finally, the supernatant was transferred to a new plastic centrifuge tube and analyzed by radio-HPLC.

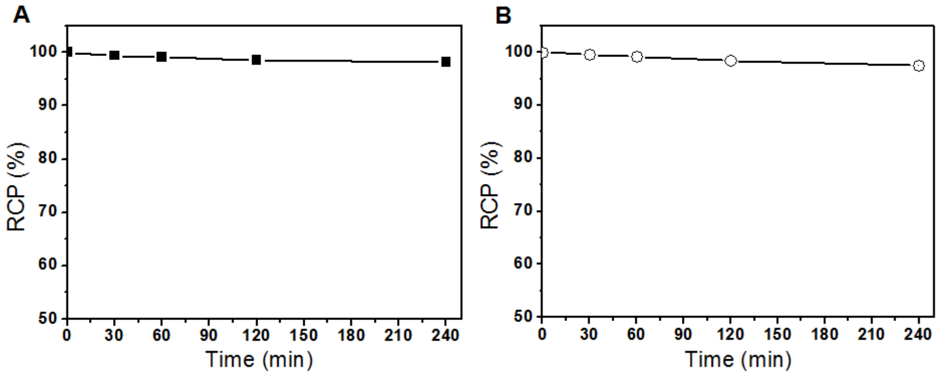

Figure S21. Stability assay of the probe [^{18}F]**1** in PBS (A) and mouse serum (B) at 37 °C for 4 h.

Western blotting analysis of caspase-3 activity in DOX-treated HeLa cells

HeLa cells were seeded into 6-well plates at the density of 5×10^5 cells/well, followed by treatment with various concentrations of DOX for 24 h. Cells were washed with ice-cold PBS and then lysed at 4 °C for 5 min with RIPA lysis buffer containing protease and phosphatase inhibitors (Halt Protease Inhibitor Cocktail, Thermo Fisher Scientific Inc.). Soluble lysates were harvested by centrifugation at 12,000 rpm for 10 min at 4 °C. The content of protein was

measured using BCA protein assay kit and 40 μg of protein was separated with a 12% SDS-PAGE gel. After blotting to PVDF membrane, the membranes were blocked with 5% skimmed milk in tris-buffered saline (TBS, pH 7.4) for 60 min at room temperature and incubated overnight at 4 °C in buffer (TBS with 0.1% Tween-20 and 5% skimmed milk) containing different antibodies. Detection of primary antibodies was performed with HRP-conjugated secondary antibody. Immunoreactive bands were visualized with Western blotting luminol reagent. Densitometric analysis was performed using Image J software.

Fluorescence imaging for localization of 1 and active caspase-3

Hela cells (20×10^5) were seeded in 1 cm glass bottom culture dishes and incubated overnight at 37 °C. Wells were divided into two groups. The control group was cultured normally for 6 h at 37 °C, then the medium was changed and the cells were cultured for another 24 h at 37 °C. The DOX-treated group was cultured in the presence of DOX (2 μM) for 6 h at 37 °C, then the medium was changed and the cells were cultured for another 24 h at 37 °C. After incubated with **1-FITC** (2 μM) for 6 h at 37 °C, the cells were washed with PBS, fixed with 3.7% formaldehyde in PBS and permeabilized with 0.1% Triton X-100 in PBS for 30 min at room temperature. Cells were blocked with 5% BSA in PBS for 30 min, and then washed with PBS. Afterwards, cells were incubated with anti-caspase-3 antibody (caspase-3 rabbit monoclonal antibody, Beyotime) overnight at 4 °C, washed with PBS, and incubated with Cy3-labeled goat anti-rabbit IgG (Beyotime) for 1 h. Cells were washed with PBS andstained with DAPI (5 μg/mL) for 2 min, and thenwashed twice with PBS. Finally, the cells were viewed with the Olympus Xcellence.

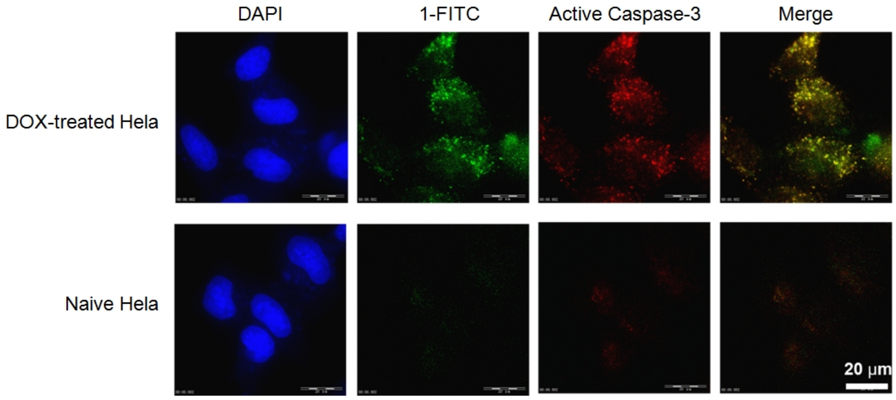

Figure S22. Fluorescence images of DOX-treated or naive Hela cells incubated with **1-FITC** (20 μM,

green fluorescence) for 6 h. The active caspase-3 was stained with anti-caspase-3 primary antibody and a Cy3-labeled secondary antibody (red fluorescence). The cell nuclei were stained with DAPI (blue fluorescence). The images of **1-FITC** and active caspase-3 were merged in the last column.

Cellular uptake assay

HeLa cells were seeded into a 6-well plate and incubated for 24 h at 37 °C. The cells were cultured in the presence of DOX (2 μM) at 37 °C for 0, 2, 4, 6, 12, and 24 h, respectively. The probe [^{18}F]**1** (1.0 μCi, 0.037 MBq) was added to each well and incubated for additional 4 h at 37 °C. For inhibition assay, the caspase inhibitor Z-VAD-FMK (50 μM) was added to the DOX-treated cells for 48 h before the addition of [^{18}F]**1**. To study the influence of cold compound **1** on cellular uptake of [^{18}F]**1** in DOX-treated or untreated cells, HeLa cells were incubated with the cold compound **1** (10 μM) and [^{18}F]**1** for 4 h at 37 °C.The cells were harvested withcentrifugal separationat 4,000 rpm for 5 min after washing twice with PBS. Then Tryp-LE™ Express (1 mL) was added and the cells were kept for 1-2 min to allow detaching. The cell/Tryp-LE solution was counted by a γ counter and the activity was normalized to the protein concentration (determined by comparison with BSA standard in an absorption assay from Pierce) in each vial.

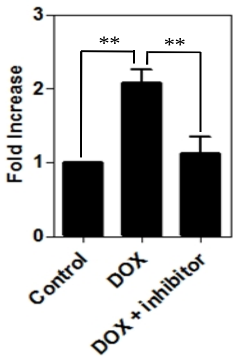

Figure S23. In vitro uptake of [^{18}F]**1** in naive HeLa cells (control), apoptotic HeLa cells induced by DOX (2 μM) and apoptotic HeLa cells treated with caspase-3 inhibitor Z-VAD-FMK (50 μM). Error bars represent standard deviation (n = 3). ** $P < 0.01$.

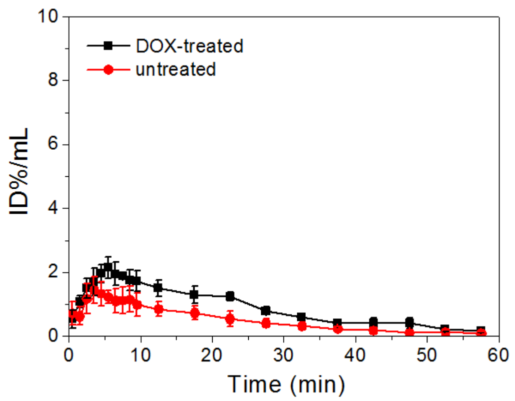

Figure S24. Time-course muscle uptake of [^{18}F]**1** in DOX-treated or untreated mice bearing Hela tumor (%ID/mL, n = 3 for each group). All the data were derived from quantification of microPET imaging data. Tissue activity was expressed as the percentage of injected dose per volume of tissue (%ID/mL). Error bars represent standard deviation (n = 3).

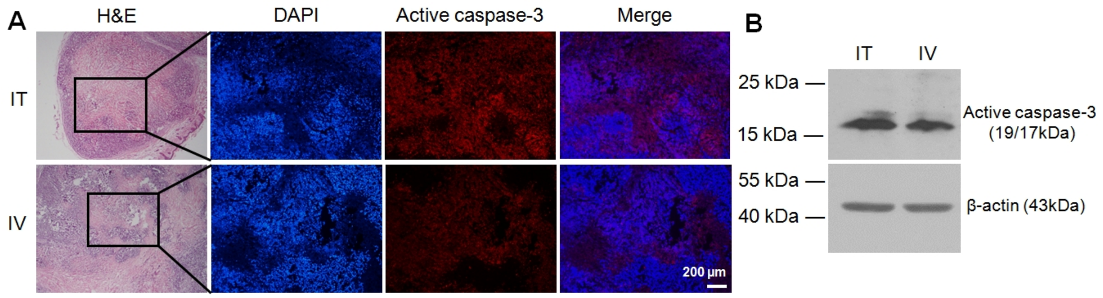

Figure S25. Histopathologic analysis of DOX-treated tumors via intratumoral (IT) and intravenous (IV) injection. (A) H&E staining and immunofluorescence staining of DOX-treated tumors via IT and IV injection. Red fluorescence showed the activated caspase-3 in DOX-treated tumor. Scale bar = 200 μm. (B) Comparison between the expression level of activated caspase-3 in DOX-treated tumors via IT and IV injection.

Table S1. Analytical HPLC condition for the compounds (**A**-**C**, **1**) (Method A).

Time (min)	Flow (mL/min)	(H_2O + 0.1 %TFA)%	(CH_3CN + 0.1 %TFA)%
0	1.0	80	20
3	1.0	80	20
35	1.0	10	90
40	1.0	80	20

Table S2. HPLC condition for the purification of **B** (Method B).

Time (min)	Flow (mL/min)	(H_2O + 0.1 %TFA)%	(CH_3CN + 0.1 %TFA)%
0	3.0	80	20
3	3.0	80	20
10	3.0	75	25
20	3.0	55	45
25	3.0	30	70
35	3.0	80	20

Table S3. HPLC condition for the purification of **1** (Method C).

Time (min)	Flow (mL/min)	(H_2O + 0.1 %TFA)%	(CH_3CN + 0.1 %TFA)%
0	3.0	50	50
3	3.0	50	50
10	3.0	20	80
15	3.0	18	82
25	3.0	15	85
30	3.0	50	50

References:

[1] Lin J, Wang W, Li K, Huang H, Lv G, Peng Y, et al. Development of a kit-like radiofluorinated biomolecule leading to a controlled self-assembly of ^{18}F nanoparticles for a smart PET imaging application. *Chem Commun.* 2017;53:6476-9.

[2] Ye D, Shuhendler AJ, Cui L, Tong L, Tee SS, Tikhomirov G, et al. Bioorthogonal cyclization-mediated in situ self-assembly of small-molecule probes for imaging caspase activity in vivo. *Nat Chem.* 2014;6:519-26.

[3] Liu Z, Pourghiasian M, Radtke MA, Lau J, Pan J, Dias GM, et al. An organotrifluoroborate for broadly applicable one-step ^{18}F-labeling. *Angew Chem Int Ed Engl.* 2014;53:11876-80.

[4] Chou TC. Derivation and properties of Michaelis-Menten type and Hill type equations for reference ligands. *J Theor Biol* 1976; 59(2):253-76.

[5] Li X, LiuQZ, Ye SQ, Wang SJ, Li K, Lv GC, et al. A protease-responsive fluorescent probe for sensitive imaging of legumain activity in living tumor cells. *Chem Biol Drug Des* 2019, DOI: 10.1111/cbdd.13530
